# Evaluating the potential of whole-genome sequencing for tracing transmission routes in experimental infections and natural outbreaks of bovine respiratory syncytial virus

**DOI:** 10.1186/s13567-022-01127-9

**Published:** 2022-12-12

**Authors:** Paul C. D. Johnson, Sara Hägglund, Katarina Näslund, Gilles Meyer, Geraldine Taylor, Richard J. Orton, Siamak Zohari, Daniel T. Haydon, Jean François Valarcher

**Affiliations:** 1 School of Biodiversity, One Health and Veterinary Medicine, University of Glasgow, Glasgow, UK; 2 HPIG. Unit of Ruminant Medicine. Department of Clinical Sciences, Swedish University of Agricultural Sciences (SLU), Uppsala, Sweden; 3 Department of Microbiology, National Veterinary Institute, SVA, Uppsala, Sweden; 4 IHAP, Université de Toulouse, INRAE, ENVT, Toulouse, France; 5 The Pirbright Institute, Pirbright, Woking, UK; 6 MRC-University of Glasgow Centre for Virus Research, Glasgow, UK

**Keywords:** BRSV, whole-genome sequencing, molecular epidemiology, cattle, transmission trees

## Abstract

Bovine respiratory syncytial virus (BRSV) is a major cause of respiratory disease in cattle. Genomic sequencing can resolve phylogenetic relationships between virus populations, which can be used to infer transmission routes and potentially inform the design of biosecurity measures. Sequencing of short (<2000 nt) segments of the 15 000-nt BRSV genome has revealed geographic and temporal clustering of BRSV populations, but insufficient variation to distinguish viruses collected from herds infected close together in space and time. This study investigated the potential for whole-genome sequencing to reveal sufficient genomic variation for inferring transmission routes between herds. Next-generation sequencing (NGS) data were generated from experimental infections and from natural outbreaks in Jämtland and Uppsala counties in Sweden. Sufficient depth of coverage for analysis of consensus and sub-consensus sequence diversity was obtained from 47 to 20 samples respectively. Few (range: 0–6 polymorphisms across the six experiments) consensus-level polymorphisms were observed along experimental transmissions. A much higher level of diversity (146 polymorphic sites) was found among the consensus sequences from the outbreak samples. The majority (144/146) of polymorphisms were between rather than within counties, suggesting that consensus whole-genome sequences show insufficient spatial resolution for inferring direct transmission routes, but might allow identification of outbreak sources at the regional scale. By contrast, within-sample diversity was generally higher in the experimental than the outbreak samples. Analyses to infer known (experimental) and suspected (outbreak) transmission links from within-sample diversity data were uninformative. In conclusion, analysis of the whole-genome sequence of BRSV from experimental samples discriminated between circulating isolates from distant areas, but insufficient diversity was observed between closely related isolates to aid local transmission route inference.

**Supplementary Information:**

The online version contains supplementary material available at 10.1186/s13567-022-01127-9.

## Introduction

Bovine respiratory syncytial virus (BRSV) is a major cause of respiratory disease in cattle. The control of BRSV has high priority due to the welfare and economic impact of this infection, and the need to decrease antibiotic use to prevent bacterial superinfections [1]. To stop BRSV circulation, vaccination and/or sanitary measures are required, neither of which are straightforward, particularly given substantial knowledge gaps around routes of transmission of BRSV. If the epidemiology of BRSV were better understood, it could facilitate the design of more effective control measures. With the goal of improving the design of control programs, currently under development in Nordic countries to improve cattle health [2], a major aim is now to identify BRSV transmission routes.

Cattle are the natural hosts of BRSV, but it is possible that other ruminant species play a role in transmission. Other animal species (e.g. birds and dogs) can act as passive vectors, although limited to certain circumstances [1]. Some data indicate that BRSV may persist in infected cattle [3–5], but attempts to demonstrate re-excretion of BRSV from previously infected animals have failed [6]. Like human RSV (HRSV), BRSV is transmitted by direct contact with infected individuals, by large respiratory droplets or nasal secretions from infected animals. Even if airborne transmission within the same building or in stables connected by a ventilation system is possible [7], it is unlikely to occur between herds over longer distances [8]. Spread of BRSV by humans acting as passive vectors, which has been observed for HRSV [9], cannot be excluded. Human visitors, who sometimes do not have direct contact with the animals, are suspected to cause some BRSV outbreaks. However, this has never been proved and more work needs to be undertaken to support this hypothesis.

BRSV is an enveloped, non-segmented, negative-stranded RNA virus that belongs to the *Orthopneumovirus* genus within the *Pneumoviridae* family [10]. The genome contains about 15 000 nucleotides and codes for 11 proteins, including a viral RNA polymerase. As with other RNA viruses, this polymerase makes errors that are not mitigated by a proofreading mechanism. For BRSV G sequence, it is estimated that the frequency of mis-incorporations per site within single field samples might be as high as 5.2 × 10^–4^–10.1 × 10^–4^ nucleotides/site [11, 12]. Therefore, nucleotide changes should be frequent, since the virus genome is relatively long and replication occurs via a positive strand intermediate. The observed frequency of nucleotide substitution varies according to gene locus, occurring preferentially in parts of the genome that tolerate changes. The BRSV G glycoprotein and SH genes have been shown to be the most variable [11, 12], however these segments are short (840 nt and 420 nt, respectively) and consequently, partial genome sequencing can fail to distinguish phylogenetic relationships of viruses collected close together in space and time [13, 14]. By studying the full genome, we hope to increase the level of genetic discrimination between strains.

Indeed, with full genome sequencing, combined with epidemiological investigations, it was possible to reconstruct transmission trees for several RNA viruses [15–19] including the foot-and-mouth disease virus (FMDV) [20–23]. Sequence data revealed substantial amounts of evolution occurring during single outbreaks [22, 23] and genetic variation was used to reconstruct transmission trees even at high spatial and temporal resolutions [20, 21].

Phylogenetic analysis of the full genome of BRSV strains might similarly enable tracing of the spread of this virus between farms and identify sources of introductions. However, to achieve this aim, it is first useful to describe the level of genetic variation in the full genome of BRSV during an infection, at the individual and herd level. Most methods for tracing routes of transmission use consensus viral genome sequences in combination with epidemiological data (e.g. time of infection, spatial proximity, contact tracing data) to weigh the relative likelihoods of potential transmission links [24]. More recently, methods for exploiting within-sample (sub-consensus) genetic diversity have been developed [25–28]. For genome sequence diversity to aid transmission tree reconstruction, there must be diversity between closely linked infected premises either at the consensus or sub-consensus level, and the amount of shared diversity between samples must be informative about their closeness on the transmission tree. In order to assess the utility of whole-genome sequences in aiding inference of transmission links between herds, we assessed the level of diversity along experimental transmission chains and between samples from recent BRSV outbreaks in Sweden at a range of scales from direct transmission between individual cattle, to samples from herds separated in space by up to 450 km. The overall goals of the study were to establish a technical approach to whole-genome sequencing of BRSV, and to determine if the spread of BRSV during an outbreak can be reconstructed from the analysis of sequencing data of the full viral genome. The objectives were to: (1) establish an NGS methodology for BRSV and evaluate its reliability; (2) gauge overall levels of diversity between (at the consensus sequence level) and within (at the sub-consensus level) samples; (3) investigate the feasibility of using consensus and sub-consensus diversity to infer transmission links; and (4) compare levels of within-sample diversity between samples, and between expected variable regions (SH + G) and less variable regions (gene F, the rest of the genome).

## Materials and methods

### Sample collection

The BRSV samples analysed in this study came from both experimental infections and natural outbreaks. Bronchoalveolar lavage, nasal swabs, and challenge virus were collected within experimental studies of calves that had been infected with BRSV, either by aerosol inhalation, or naturally (Table 1). In total, 36 samples from 19 animals experimentally infected with one of four different strains were sequenced. In addition, six challenge virus samples consisting of four different strains obtained by cell culture or calf passage were sequenced (Table 1) [29–33]. Nasal swabs were collected from the nostrils of live animals, whereas bronchoalveolar lavage (BAL) was obtained post-mortem by rinsing the left lung lobes with 400 mL phosphate buffered saline (PBS) through the trachea. The BAL was filtered through a sterile gauze and 10 mL were centrifuged at room temperature and 200 × *g* for 10 min. The supernatant was discarded and the pellet was resuspended in 750 µL TRIzol™ Reagent (Thermo Fisher, Life Technologies Corporation, USA) and frozen at −75 °C until RNA extraction. Furthermore, seven nasal swab samples were collected from BRSV outbreaks in five herds in 2020.

**Table 1:** **Experiments and studies from which samples originated**

Study	Year (reference)	Infection method	Challenge virus^a^
A	2002 [29]	Experimental aerosol	BRSVDKp7: Cell lysate containing DK 9402022 obtained by isolation and seven passages in FBL [35]
B	2010 [30]	Experimental aerosol	BRSVDKp7: Cell lysate containing DK 9402022 obtained by isolation and two passages in FBL and five passages in FBT [35]
C	2012 [31]	Experimental aerosol	BRSVSnook: BAL containing the BRSV Snook strain obtained by isolation in CK followed by three passages in gnotobiotic calves [36]
D	2010 [32]	Experimental intranasal/intratracheal	BRSV3761p2: Cell lysate containing BRSV-3761, obtained by isolation in FBT followed by three passages in newborn calves [32]
F	2018 [33]	Experimental aerosol	Same as experiment C
G	2018 (This work)	Experimental aerosol	BRSV/Sweden/HPIG-SLU-620-Lovsta/2016 p4: Cell lysate containing BRSV-SE2016-620 obtained by isolation and four passages in FBT, or BAL containing the same strain that had been passaged once or twice in calves
O	2020 (This work)	Natural outbreak	N/A

*FBL* foetal bovine lung cells, *FBT* foetal bovine turbinate cells, *BAL* bronchoalveolar lavage, *CK* calf kidney cells.

### Experimental samples

Experiments A to F were previously published experimental BRSV infections of calves that were conceived for evaluation of different vaccines [29–33]. Within these studies, BAL were collected post-mortem as described above and/or daily nasal swabs were collected from one nostril of the calves before and after BRSV challenge. The challenge consisted of virus passaged in cell culture or calves, as described in Table 1.

**Figure 1:**
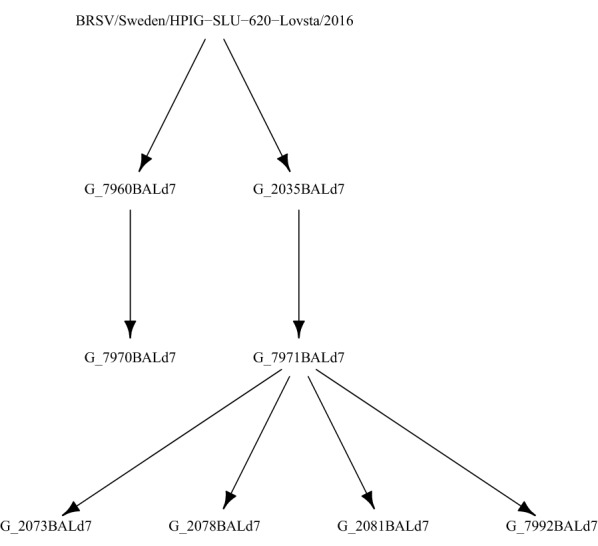
**Schematic outline of Experiment G.** A BRSV strain (BRSV/Sweden/HPIG-SLU-620-Lovsta/2016) was isolated from a conventional calf in a natural BRSV outbreak and used to inoculate two calves (7960 and 2035), by aerosol. Bronchoalveolar lavages obtained on 7 days post-infection from these calves were used to inoculate calves 7970 and 7971, respectively. Bronchoalveolar lavage was obtained on 7 days post-infection from calf 7971 was then used to inoculate four new calves. The calves were separated in isolated pens (together with immunised companion calves), to avoid cross-contamination between the animals.

Experiment G included passage of virus between calves and was designed with the aim of studying the evolution of the BRSV genome along serial passages (Figure 1; Additional files 1A, D). Ten calves were obtained from the Swedish Livestock Research Centre (SLU, Uppsala). They were conventionally reared 3–7 week-old male and female calves of Swedish Holstein and Swedish red and white breed and were selected based on having no history of respiratory disease or vaccination. The calves were housed in pairs in straw-bedded pens and were fed milk replacer as well as calf pellets twice daily, in addition to hay and water *ad lib*. After a week of acclimatisation, calves 7960 and 2035 were challenged with 1.84–2.35 × 10^5^ equivalent TCID_50_ of a BRSV strain (BRSV/Sweden/HPIG-SLU-620-Lovsta/2016) by aerosol inoculation, as determined by realtime quantitative PCR [31]. Daily nasal swabs (Virus swab UTM™, Copan, Italy) were collected from post-infection day (PID) 0 to PID 7 or 10. Before and after challenge, daily clinical examinations were performed and the clinical signs of respiratory disease were scored, as described previously [31]. Clinical and virological data are shown in Additional file 1E–G. Seven days post-challenge, calves 7960 and 2035 were euthanized under general anesthesia overdose (5 mg/kg ketamine followed by 40 mg/kg pentobarbital sodium and exsanguination) and lungs were excised for observations and quantification of the macroscopic lesions by observation and palpation, and for collection of BAL. This procedure was repeated twice over two further rounds of passage. BAL obtained from calves 7960 and 2035 was used to inoculate calves 7970 and 7971, respectively. BAL obtained on PID 7 from calf 7971 was then used to inoculate calves 2073, 2078, 2081 and 7992, and BAL was obtained from these four calves on PID 7 (Additional file 1A, D). Whole genome sequence was generated from these eight calves. The remaining two calves (7965 and 7962) served as companion animals. Virus from these animals was not used for further inoculation or for genome sequencing.

### Outbreak samples

**Figure 2:**
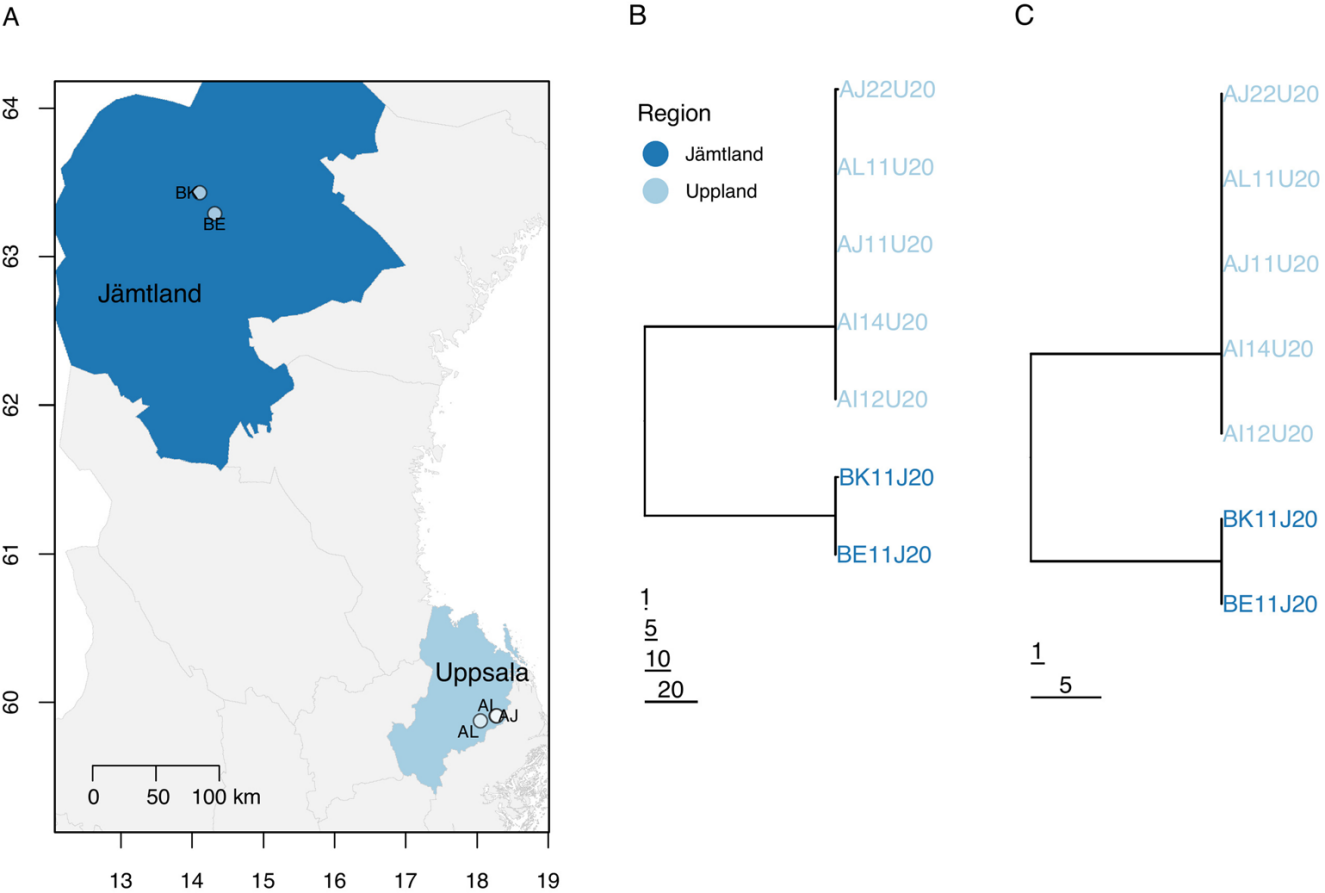
**Geographic location and phylogenetic tree of the field outbreak samples.** Map showing the location of the two farms in Jämtland (BE and BK) and three in Uppsala (AI, AJ and AL) from which the seven field outbreak samples were collected (**A**). Neighbour-joining phylogenetic trees of the consensus sequences estimated from the seven field samples using whole-genome sequences (**B**), and only the region including genes SH and G (**C**). Trees were rooted at their midpoints. Horizontal branch lengths indicate the number of sites that differed between sequences.

The field outbreak samples (study O) were nasal swabs collected from cattle with respiratory disease in three farms in Uppsala county (farms AI, AJ and AL, sampled on the 18th, 19th and 21st February 2020, respectively) and two farms in Jämtland county that were sampled approximately seven weeks later (farms BE and BK, sampled on the 3rd and 13th April 2020, respectively) (Figure 2A). Farms AI and AJ were situated 500–1000 m from each other, and 13 km from AL, while BE and BK were situated 19 km apart. The counties of Uppland and Jämtland are separated by approximately 450 km. Both previously unpublished studies (G and O) were approved by the Ethical Committee of the University of Uppsala, Sweden (Ref. no. 5.8.18–16188/2017). The BRSV strains were sequenced directly from nasal secretions without cell culture passage.

### Sample nomenclature

All sample names presented here are prefixed with the experiment letter (A, B, C, D, F or G) or the letter O for the outbreak samples. Samples taken from experimental animals are named as follows: [*experiment letter*]_[*4-digit animal ID*][*sampling method: BAL (bronchoalveolar lavage) or NS (nasal swab)*]d[days post-infection]. E.g. B_8863NSd6 was sampled as part of experiment B from animal 8863 by nasal swab at 6 days post-infection. Outbreak samples are named as follows: O_[*two-letter anonymised farm code*][*two-digit animal code*][*county code:* U *for Uppsala;* J *for Jämtland*][*two-digit year of sampling*]. E.g. O_AJ22U20 is an outbreak sample taken on farm AJ from animal 22 (meaning that on the second visit, this was the second animal sampled) in Uppsala in 2020.

### Sequencing

Total RNA was extracted from clinical samples using a combination of TRIzol (Invitrogen, Carlsbad, CA, USA) and RNeasy columns (Qiagen, Hilden, Germany). The RNA was converted into cDNA by using Superscript IV First-Strand Synthesis Kit (Invitrogen). The concentration of each sample was determined using High Sensitivity reagents with Qubit 2.0 fluorometer (Invitrogen). In order to reduce bias, there was no PCR amplification step. However, samples were analysed by RT-qPCR in parallel and only samples with CT values below 25 were included. DNA libraries were prepared using the NexteraXT kit (Illumina, San Diego, CA, USA) according to the manufacturer’s instructions. Each DNA sample was initially analysed by Bioanalyzer (Agilent Technologies, using DNA1000 chips) to ascertain library quality and average size distribution. DNA libraries were sequenced on the Illumina MiSeq in paired-end mode using the MiSeq 600 cycle v3 kit (Illumina). Raw sequence data were analysed and mapped using Genomics software package, workbench 10 (CLC Bio, Aarhus, Denmark). Raw reads were trimmed, and subsets of each trimmed dataset were assembled de novo to generate reference sequences for each data set. Basic trimming was done by CLC-bio software to generate FastQ files. The FastQ files were deposited in the NCBI Sequence Read Archive database (accession numbers are provided in Additional file 1B) and are collected under BioProject PRJNA893434.

### Bioinformatics pipeline

Adaptor trimming and quality control of paired-end reads was performed on the FastQ files using *Trim Galore* version 0.6.6 [34]. Reads shorter than 50 bp were discarded and low-quality ends with a Phred quality score below 25 were trimmed. The trimmed reads from the challenge virus samples from each experiment were aligned to a closely related reference sequence [32, 35, 36] using *BWA* version 0.7.17 [37] with the MEM algorithm and the default *BWA* options. All the experimental samples were then aligned to the consensus sequence of the challenge virus specific to that experiment, with the BWA gap-opening penalty increased from the default of 6 to 100 to prevent insertions. This ensured that the within-host diversity data generated by the pipeline was suitable for the downstream diversity analyses that do not allow insertions or deletions (specifically *Betabinomial Bottleneck* and *BadTrIP*). The field outbreak samples were aligned to the challenge strain from experiment G, BRSV/Sweden/HPIG-SLU-620-Lovsta/2016, which was isolated from a natural infection sampled in Uppland in 2016. Preliminary analysis showed that coverage within the first ~500 bases tended to be unusually low. We therefore excluded gene NS1 (bases 1–572) and the sequence between NS1 and NS2 (573–578) from the reference sequences, and started aligning at the start of NS2 (base 579). Consequently, we analysed 96% of the BRSV genome; however, for brevity we refer to these sequences as whole genome sequences. Following conversion to bam alignment format using *samtools* version 1.9 [38] consensus sequences and per-genome position base counts were produced using *VSensus* [39].

### Statistical analysis

#### Sequence quality analyses

We conducted three preliminary analyses with the aim of predicting and potentially increasing coverage. First, we used linear mixed-effects regression models implemented in the *lme4* package for *R* version 4.1.1 to investigate the effect of sampling method. We asked if high-coverage genome sequences could be achieved using nasal swab samples, or if it were necessary to use the more intrusive and labour-intensive BAL method. The outcome variable was coverage, log_10_-transformed to satisfy the assumption of normally distributed homoscedastic residuals. We used matched samples (a sample using one method was included only if another sample had also been collected by the other method from the same animal on the same day) and included a random effect for sampling event (animal-day). Four animals were sampled by both bronchoalveolar lavage and nasal swab, yielding a total of eight bronchoalveolar lavage samples and eight nasal swab samples. Second, we used a linear mixed-effects model to assess the utility of real-time PCR (RT-PCR) Ct values for predicting mean coverage, with the aim of using Ct value to focus sequencing resources on high coverage samples. The model was adjusted for the effect of sampling method (bronchoalveolar lavage or nasal swab) and included a random effect for sample ID to account for repeated sequencing of the same sample. The proportion of variance in log_10_ coverage explained by Ct value was estimated as marginal *R*^2^. Seventy-one sequences of 40 samples were included in this analysis. Third, we conducted a paired t-test of log_10_ mean coverage of 50 samples to ask if high-coverage sequence that is informative about within-sample diversity could be recovered from low-coverage sequences by focusing on the 1344-nucleotide sequence containing genes SH and G (including its 39-nucleotide intergenic sequence), which comprises 9% of the genome and is expected to be its most variable region [11, 12]. We refer to this as the SH + G region.

#### Reliability of viral diversity data

To give sufficient resolution to assay low frequency polymorphisms, we excluded from within-sample diversity analyses sequences with mean coverage below 500 × or where the 10th centile of coverage was below 125 × . This second criterion was intended to ensure consistency of coverage, but in practice all samples that met the first criterion also met the second. We refer to this as the 500 × coverage data set. The sequences that passed these two thresholds included ten samples that had been sequenced more than once (six twice and four three times). We used a binomial generalised linear mixed-effects model implemented in the *lme4* package to assess the reliability of the sequencing methodology and to choose the error frequency threshold of 1% below which polymorphisms were likely to be artefacts of the sequencing process. For this analysis we defined polymorphisms as minority variants. That is, all non-majority bases were pooled and counted as mismatches, and the mismatch proportion at genome site *i* from sample *j* (*j* = 1, …, *k*) was defined as *p*_*ij*_ which was estimated as *x*_*ij*_/*n*_*ij*_ where *x*_*ij*_ is the total number of mismatches and *n*_*ij*_ is the coverage. In order to find the optimal error threshold, we modelled mismatches at site *i* from sample *j* as arising from a binomial distribution, *x*_*ij*_ ~ Binom(*n*_*ij*_, *p*_*ij*_). We modelled both inter-site variation in mismatch frequency and error variation as normally distributed deviations on the logit scale: logit(*p*_*ij*_) = *μ*_*j*_ + γ_*i*_ + ε_*ij*_, where *μ*_*j*_ is the mean logit mismatch frequency across all sites in sample *j*, γ_*i*_ ~ N(0, σ_γ_^2^) represents true mismatch variation among sites, and ε_*ij*_ ~ N(0, σ_ε_^2^) represents extra-binomial error variation not shared between the repeat sequenced samples. We defined the correlation between repeat sequences of the same sample as a latent-scale intra-class correlation coefficient, ICC = σ_γ_^2^/(σ_γ_^2^ + σ_ε_^2^). The frequencies of true polymorphisms should be highly correlated between repeat sequences of the same sample, while error variation should be uncorrelated. For each set of repeat-sequenced samples, we estimated ICC from *x*_*ij*_ and *n*_*ij*_ from 20 data sets with successively more stringent error thresholds. In the first data set, sites with a mean mismatch frequency below 0.1% were removed, with this error threshold being increased to 2.0% in steps of 0.1%. Error thresholds from comparable data are commonly in the range 0.5–1.0% [40, 41]. We reasoned that the ICC should increase as the error threshold increases and plateau once the correct error threshold has been passed. In order to detect any artifactual tendency for ICC to plateau at higher error thresholds, we estimated ICC from three data sets where site identity had been randomly permuted to abolish the correlation between the two samples (expected ICC = 0). Variant frequencies below the 1% threshold selected using this analysis were assumed to be errors and set to zero.

#### Consensus-level diversity

Consensus sequences were called after excluding sequences with mean coverage below 20 × or where the 10th centile of coverage was below 5 × (although, as with the diversity analysis, all samples that met the first criterion also met the second). We refer to this as the 20 × coverage data set. Diversity between consensus sequences within each experiment and between the field samples was measured by the number of segregating sites. To illustrate the genetic structure of the field outbreak samples, an unrooted neighbour-joining (NJ) phylogenetic tree was constructed, using genetic distance measured by the numbers of sites that differed between each pair of consensus sequences. We re-made the NJ tree using only the SH + G region to test whether an equally informative phylogenetic tree could be obtained by partial sequencing.

#### Within-sample diversity

Within-sample diversity at each site was quantified as the frequency of the minority variant (including only variants with frequencies above the 1% error threshold). That is, all non-majority bases were pooled and counted as mismatches, and the mismatch frequency at site *i* (*i* = 1, …, *N*) was defined as *p*_*i*_ estimated as *x*_*i*_/*n*_*i*_ where *x*_*i*_ is the total number of mismatches and *n*_*i*_ is the coverage at site *i*. Diversity within samples was estimated using Shannon diversity, $H=-\sum_{i=1}^{N}{p}_{i}\mathrm{ln}({p}_{i})$, where $0\times \mathrm{ln}(0)$ was taken to be zero. The distribution of diversity along the genome was examined by plotting per-site Shannon diversity, defined as the contributions of individual sites to Shannon diversity. We also estimated diversity as the proportion of sites with mismatch frequency above the 1% error threshold. To investigate whether the greater diversity of the SH + G region at the consensus level extended to sub-consensus diversity, both diversity measures in each sample were compared between SH + G and gene F which is expected to be less variable than SH + G based on previous studies [11, 12] and between SH + G and the whole BRSV genome.

#### Bottleneck size and transmission tree distance

We hypothesised that within-sample diversity could assist inference of transmission links. We tested this hypothesis using two methods that exploit within-sample diversity while tolerating sequencing errors: a population genetic model-based method that estimates the size of a transmission bottleneck [42]; and *BadTrIP*, a Bayesian transmission tree reconstruction method [26].

We applied the bottleneck method to the experiment G samples, where the transmission tree is known (Figure 1), by estimating the correlation between pairwise transmission tree distance and pairwise shared genetic diversity, using only samples that passed the 500 × coverage threshold. If within-sample diversity is informative about the transmission tree, we would expect a negative correlation between the number of transmission steps between each pair of samples, and the amount of genetic diversity they share. The exact beta-binomial method was used to estimate bottleneck size and its 95% confidence interval (CI), implemented in an R script from Sobel Leonard et al. modified to reduce computation time [43]. Bottleneck size can be interpreted as the number of individual virions from an infection donor that established a new population in the recipient. It is a measure of shared genetic diversity because the amount of genetic diversity transferred through a transmission bottleneck is proportional to its size. If bottleneck size is estimated between donors and recipients separated by multiple transmission steps, repeated bottlenecks of similar size are expected to act cumulatively, leading to the expectation of a negative correlation between transmission tree distance and perceived bottleneck size. We also estimated bottleneck size between all pairs of field outbreak samples that passed the 500 × coverage threshold. While for experiment G we estimated bottleneck size in the known transmission direction only, for the field outbreak samples the direction of transmission was unknown, or is not applicable, so we estimated bottleneck size in both directions. In the absence of a known transmission tree, we used spatial and temporal proximity and contact tracing data to assign an ordinal “epidemiological distance” category: (1) same farm; (2) different farms with strong evidence of contact; (3) different farms with moderate evidence of contact; (4) different farms with no evidence of contact.

The *BadTrIP* method uses Monte Carlo Markov chain (MCMC) to infer the posterior distribution of transmission trees from genomic diversity data in the form of the four observed base frequencies at each site. *BadTrIP* is implemented as a package (*BADTRIP* v1.0.1) in *BEAST* v2.5.2 [44] and was applied to samples from experiment G and to the field outbreak samples. In order for the Monte Carlo Markov chain (MCMC) to converge on a timescale of days rather than several weeks, it was necessary to analyse subsets of only two to five samples. For the same reason of computational feasibility, coverage was stochastically down-sampled to a maximum of 50 × . With these adjustments, *BadTrIP* converged in an average of four (range 1–20) days, where convergence was defined as exceeding an effective sample size (ESS) of at least 200 from the posterior model probability and at least 100 from all parameters. In order to isolate the contribution of genetic data to transmission tree reconstruction, identical uninformative epidemiological data were provided to *BadTrIP* for each sample, with all sampling times set to day zero and all exposure windows set to ± 100 days. The virtual population size of the PoMo model was set to 25, the sequencing error rate was estimated rather than fixed, the base frequencies were fixed to the mean sample frequencies, and the F81 substitution model [45] was assumed. The mean and standard deviation of the lognormal prior distribution of bottleneck size were 0 and 2 respectively.

## Results

### Sequence quality analyses

Mean coverage in NS samples (*n* = 8) was 10.7-fold higher (95% CI: 3.9, 35.3; χ^2^_df=1_ = 14.41, *P* = 0.0001; marginal *R*^2^ = 54%) than in BAL samples (*n* = 8) taken from the same four animals (Additional file 1H), therefore BAL does not convey a coverage advantage to offset the greater costs of sampling. Coverage was negatively associated with Ct value in NS samples (*n* = 37 sequences; χ^2^_df=1_ = 4.71, *P* = 0.030) but there was no evidence for an association in BAL samples (*n* = 34 sequences; χ^2^_df=1_ = 0.29, P = 0.59; interaction χ^2^_df=1_ = 4.78, *P* = 0.029). However, the proportion of variance in log-transformed coverage explained by Ct variation was low within both sample types (0.5% with BAL and 11.3% with NS), indicating that Ct value is not a strong predictor of coverage regardless of sampling method. Mean coverage in the SH + G region was 13% higher than mean coverage across the whole genome (geometric means 349 × and 310 × respectively; paired t-test of log_10_ mean coverage: *t*_df=49_ = 2.09, *P* = 0.042), but in low-coverage samples (coverage < 500 ×) there was no difference (the geometric means were 92 × and 83 × respectively; *t*_df=29_ = 1.46, *P* = 0.156). These results suggest that samples with low coverage overall will also have low coverage within the SH + G region and are therefore unlikely to be rescued by focusing on this region.

### Reliability of viral diversity data

Analysis of repeat sequences of the same samples indicated that the sequencing methodology gave reliable results for polymorphisms at a frequency above approximately 1% frequency. In nine of the ten samples that had been sequenced more than once, the relationship between ICC and error threshold broadly followed the expected pattern, rising from 0.4 to 0.8 at the lowest error threshold to a plateau that was above 0.9 in seven of the nine samples and around 0.7 in the other two (Additional file 1I). This pattern suggests that, as expected, lower frequency polymorphisms are less reliable and therefore more likely to be errors, and that the sequencing methodology is reliable. FastQ files from repeat sequences were therefore merged, yielding the final diversity analysis data set consisting of sequences of 16 experimental samples and 4 field outbreak samples with coverage of at least 500 × and with polymorphisms at frequencies below an error threshold of 1% set to zero (Table 2).

**Table 2:** **Numbers of hosts sampled, sequencing coverage statistics, and numbers of samples sequenced from each experiment**

Sample source	N animals sampled	Mean (range) coverage	Coverage threshold	N samples sequenced				N variable sites (cov. ≥ 20 ×)
				Challenge virus	BAL	NS	Total	
Expt A	0	5234 (−)	< 20 20–500 ≥ 500	0 0 1	0 0 0	0 0 0	0 0 1	–
Expt B	2	2323 (64, 8614)	< 20 20–500 ≥ 500	0 0 1	0 2 0	0 2 1	0 4 2	0
Expt C	2	1952 (148, 7925)	< 20 20–500 ≥ 500	0 1 0	0 1 1	0 1 2	0 3 3	1
Expt D	2	108 (53, 207)	< 20 20–500 ≥ 500	0 1 0	0 2 0	0 1 0	0 4 0	1
Expt F	5	604 (1, 2195)	< 20 20–500 ≥ 500	0 0 1	2 1 0	0 9 3	2 10 4	7
Expt G	8	1314 (65, 5856)	< 20 20–500 ≥ 500	0 0 1	0 3 5	0 0 0	0 3 6	0
Outbreak	8	1700 (12, 7691)	< 20 20–500 ≥ 500	– – –	0 0 0	1 3 4	1 3 4	146
Total	27	1413 (1, 8614)	< 20 20–500 ≥ 500	0 2 4	2 9 6	1 16 10	3 27 20	–

The numbers are broken down by coverage threshold (< 20 × : not used; 20–500 × : suitable for consensus sequence analysis only; ≥ 500 × : suitable for both consensus sequence analysis and within-host diversity analysis) and by sample type (challenge virus, bronchoalveolar lavage [BAL] or nasal swab [NS]). The number of segregating sites among consensus sequences is also presented.

### Consensus-level diversity

Of the 50 sequenced samples collected from 27 animals and six challenge viruses, 47 sequences from 26 animals and six challenge viruses passed the coverage criteria for consensus sequences (the 20 × coverage data set). The 47 consensus sequences were deposited in the NCBI Nucleotide database (accession numbers are provided in Additional file 1B). The distribution of coverage along the genome for each sample and the proportion of reads that mapped to the reference sequence (mean: 3.8%; range: 0.1–24.0%) are reported in Additional file 2. Very low levels of consensus-level diversity, measured as number of segregating sites, were found in the five experiments (B, C, D, F and G) that yielded more than one sequenced sample (Table 2; Additional file 3). No consensus-level changes were observed in experiment B, between the challenge virus and five samples taken from two calves 4–6 days post-infection; or in experiment G, between the challenge virus and eight samples taken up to 21 days and three transmission steps after the initial infection. In experiment C there was one consensus-level polymorphism relative to the challenge virus, which was found in three of the six samples, and both calves (Table 3). This polymorphism also differed between two samples taken on the same day from the same animal by different methods, BAL and NS. However, the minority variant frequencies at this site in the six experiment C samples were close to the consensus threshold of 50% (range 39.6–49.6%), therefore consensus sequence differences between these samples reflected a narrow spectrum of differences in diversity between the viral populations at this site. In experiment D, one consensus-level change relative to the challenge virus was observed in both samples from one animal but not the single sample from the other animal. Both animals in experiment D were infected directly from the challenge virus. The same consensus-level change that was seen in experiment C, at amino acid 305 in gene L, was also observed in experiment F, but in the opposite direction (Table 3). This was the only site that differed in consensus sequence between the 13 experiment F samples collected from five hosts at 4–7 days post-infection, being present in the consensus sequence of three samples from two calves. Six other sites differed between the host samples and the challenge virus but not between the host samples. The sub-consensus diversity underlying the consensus-level variation observed in the experimental samples revealed a common pattern. All the consensus-level variants observed in the calves in experiments C, D and F were present at sub-consensus frequencies ranging from 15 to 40% in the challenge virus, suggesting that the consensus level diversity arose from pre-existing genetic variation in the challenge virus, rather than from new mutations.

**Table 3:** **Characteristics of variation among consensus sequences among experimental samples**

Experiment	Genome position	Gene	Gene position	A.a. position	Codon/base change	A.a. change	Freq. in challenge
C	9328	L CDS	922	305	GAC → GTC	Asp → Val	39.6%
D	3413	M CDS	203	65	CTA → CCA	Leu → Pro	14.9%
F	589	NS2 5′-UTR	9	–	C → A	–	14.9%
F	4855	G CDS	166	51	ATG → TTG	Met → Leu	17.5%
F	5819	F CDS	263	84	AAA → GAA	Lys → Glu	40.0%
F	6630	F CDS	1074	354	CTG → CAG	Leu → Gln	20.9%
F	7651	M2 CDS	138	43	CTG → CTA	Synonymous	23.3%
F	9328	L CDS	922	305	GTC → GAC	Val → Asp	16.0%
F	12089	L CDS	3683	1226	AAT → GAT	Asn → Asp	31.1%

Nucleotide position and amino acid (a.a.) position are relative to NCBI Reference Sequence NC_038272.1 (BRSV ATCC51908 complete genome). Codon, base and a.a. changes are relative to the experimental challenge virus consensus sequence.

CDS: coding sequence, UTR: untranslated region.

In contrast to the 40 experimental samples, consensus-level diversity between the seven field samples was relatively high with 146 segregating sites observed. Diversity was strongly partitioned between the two Jämtland herds (BE and BK) and the three Uppland herds (AI, AJ and AL), with 144/146 polymorphisms being region-specific (Figure 2B; Additional file 3). There was only one consensus-level difference between herds within each region.

Consensus-level diversity was higher in the SH + G region than across the whole genome, as expected: 27/1344 (2.0%) sites varied between the seven field outbreak sequences, while across the whole genome only 146/14 562 (1.0%) sites varied. The SH + G region therefore captures 18% of consensus sequence diversity in only 9% of the genome. In addition, the whole-genome and SH + G NJ trees were highly similar (Figures 2B and C).

### Within-sample diversity

**Figure 3:**
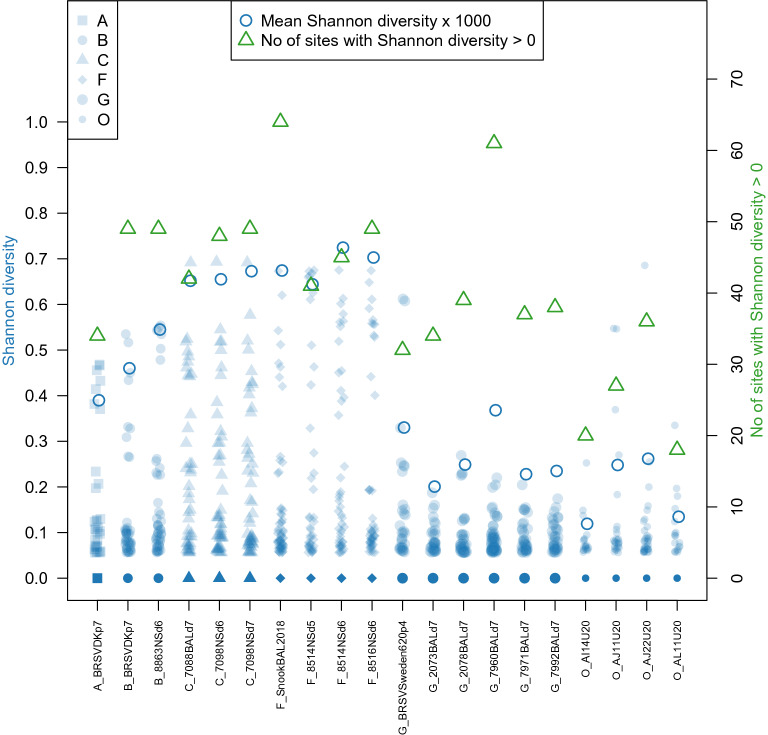
**Within-sample diversity in the 20 samples from the 500 × coverage sequence set.** Three measures of diversity are plotted: Shannon diversity at each site (blue shaded points, left axis); mean Shannon diversity across the genome (open blue circles, left axis), multiplied by 1000 to allow plotting on the same scale as site-specific Shannon diversity; and the number of sites with Shannon diversity > 0 (green triangles, right axis). Base proportions less than 1% were assumed to be due to NGS error and set to zero, and therefore did not contribute to diversity estimates. The mean number of sites for each sample was 14 562 (range 14 561–14 563). The challenge virus from experiment G, BRSV/Sweden/HPIG-SLU-620-Lovsta/2016, is abbreviated to G_BRSVSweden620p4.

Diversity within the 20 sequences in the 500 × coverage data set was quantified using Shannon diversity and the number of diverse sites (Figure 3). In contrast to consensus-level diversity, within-sample diversity tended to be higher in the experimental samples than in the outbreak samples, although diversity in experiment G was lower than in the other experiments and close to the levels of the field samples. The number of sites with sub-consensus diversity above the error threshold of 1% ranged from 32 to 64 (0.2–0.4% of the genome) in the experimental samples and from 18 to 36 (0.1–0.2% of the genome) in the field outbreak samples (Figure 3, green triangles). Higher frequency diversity was particularly scarce in the five experiment G samples in the 500 × coverage data set, with no sites in any sample having a mismatch frequency above 10%, although there were three such sites in the challenge virus sample. Higher levels of common within-sample variation were found in experiments A, B, C and F, with between six and 12 sites per sample above the 10% mismatch frequency threshold, and intermediate in the four field outbreak samples (range 0–4 sites with > 10% mismatch frequency).

**Figure 4:**
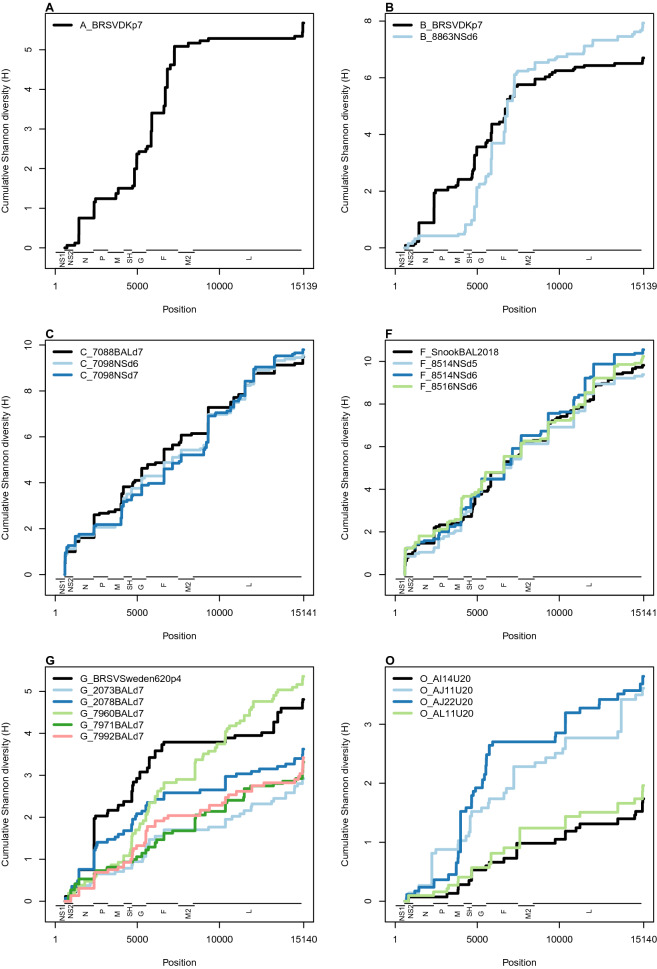
**Cumulative Shannon diversity along the BRSV genome.** Cumulative Shannon diversity along the BRSV genome is plotted for each of the 20 samples in the 500 × data set. A uniform distribution of diversity along the genome would produce a straight diagonal line. Base proportions less than 1% were assumed to be due to NGS error and set to zero. Gene positions are shown at the foot of each plot. Due to the omission of positions 1–578, including all of gene NS1, from sequencing (see Materials and methods), cumulative diversity begins at 579, at the 5’ end of gene NS2. The challenge virus from experiment G, BRSV/Sweden/HPIG-SLU-620-Lovsta/2016, is abbreviated to G_BRSVSweden620p4.

The cumulative distribution of Shannon diversity is shown in Figure 4. In experiments A and B, the highest diversity was between positions 5000 and 7500, a region approximately spanning the G, F and M2 genes. In experiments C and F, diversity was uniformly distributed along the genome. The regions of highest diversity in experiment G were approximately 2000–6000, including genes P, M, SH and G. Diversity was approximately uniformly distributed along the genome in three of the four field outbreak samples (AI14U20, AJ11U20, AL11U20). The region of highest diversity in the remaining field sample, AJ22U20, was between positions 4000 and 6000, covering genes SH and G. Several sequences from experiments A, B and G and from the field outbreak showed a sharp spike in diversity around positions 15 050–15 100 in the 3’ untranslated trailer region.

**Figure 5:**
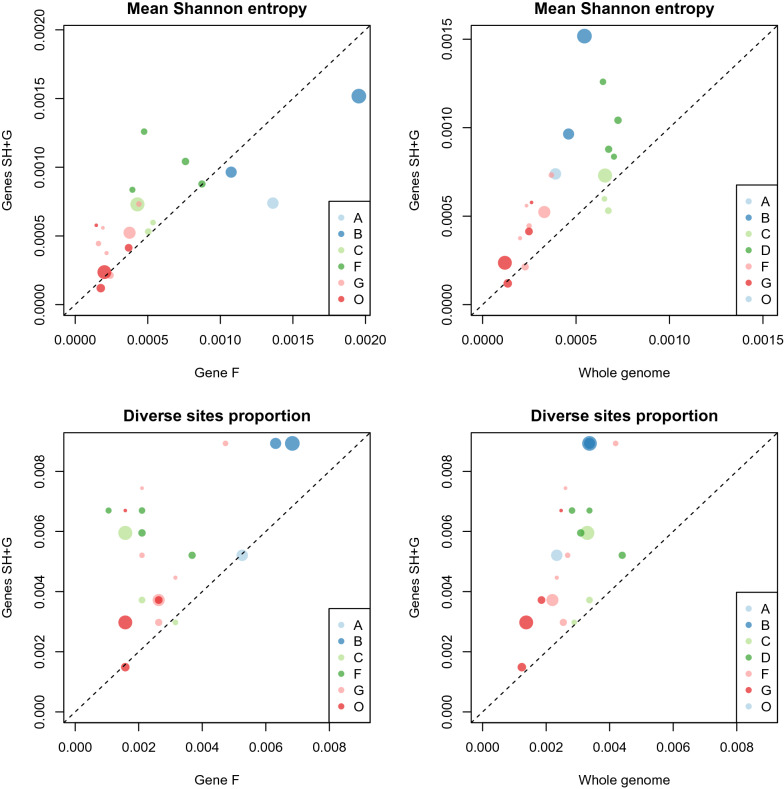
**Within-sample diversity in genes SH and G.** Comparison of within-sample diversity in genes SH and G with gene F (left) and with the whole BRSV genome (right) from each experiment (A, B, C, F and G) and in the field outbreak samples (O). Diversity is measured as the mean per-site Shannon diversity (top row) and the proportion of sites with diversity above the 1% error threshold (bottom row). Each point represents a sample, with samples above and to the left of the diagonal showing higher diversity in the SH + G region. Point area is proportional to coverage.

Comparison of diversity between genes SH and G, where diversity was hypothesised to be concentrated, and gene F showed that for the majority of experimental and field outbreak samples, mean Shannon diversity was higher, and there were more diverse sites, in genes SH and G than in gene F (Figure 5). The main exceptions to this pattern were experiments A and B, where diversity was higher in gene F. The tendency towards showing higher diversity in genes SH and G was even more pronounced when compared with diversity averaged across the whole BRSV genome.

### Bottleneck size and transmission tree distance

**Figure 6:**
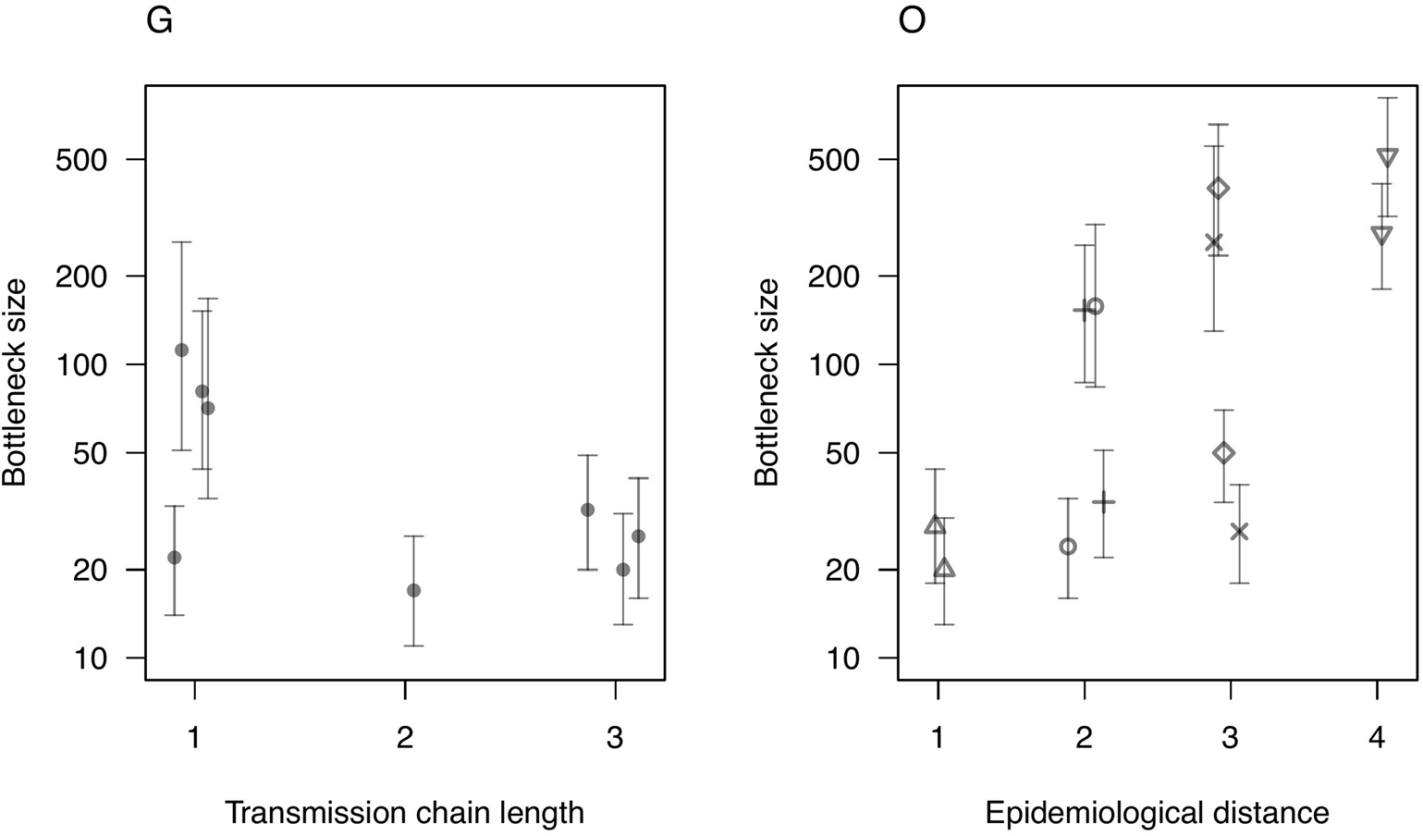
**Estimated sizes of transmission bottlenecks.** Estimated transmission bottleneck size (± 95% CI) is plotted against transmission chain length for experiment G and against epidemiological distance for the field outbreak samples (O). Because the direction of transmission was not known for the field outbreak samples or not applicable, bottleneck size was estimated in both directions, with common point shapes indicating pairs of opposite-direction bottleneck estimates between the same samples.

Six samples from experiment G, and four samples from the field outbreak, all of which were collected in Uppland within a four-day period, passed the 500 × coverage threshold and were therefore suitable for estimation of transmission bottleneck sizes (Figure 6; Additional file 1C). Bottleneck sizes between the six animals from experiment G ranged from 17 to 112 virions, while the six bottleneck sizes estimated in each direction between the four field outbreak samples ranged from 20 to 516. Due to the small number of samples that passed the 500 × coverage threshold, it was not possible to make inferences about the relationship between transmission tree distance and bottleneck size.

No high confidence transmission links were inferred between samples from experiment G and from the field outbreak using *BadTrIP*, with the highest confidence link being the inference of the direction of transmission from farm BE to farm BK with 68% probability, which coincided with the direction of transmission suspected based on contact tracing; that is, the suspected direction of transmission was estimated to be approximately twice as likely as the opposite direction (Table 4). For the remainder of the field outbreak samples and for experiment G, all possible trees were found to be approximately equally probable. Table 4 summarises the results of the *BadTrIP* analyses alongside evidence from consensus sequences and, for the field outbreak samples, contact tracing, while the detailed *BadTrIP* results are shown in Additional files 1J and K.

**Table 4:** **Assessment of epidemiological and genomic evidence for transmission among animals from experiment G and from the field outbreak**

Samples	Description of contact	Consensus-level and within-sample diversity	Probability of transmission inferred from within-sample diversity
Experiment G: G_2035, G_7971, G_2073, G_2078, G_7992	Experimental transmission from G_2035 to G_7971 and from G_7971 to G_2073, G_2078 and G_7992	No consensus-level diversity	No transmission link inferred with high confidence (max. probability 35%)
Field outbreak: BE11J20, BK11J20	Day 0: One cattle, which did not develop clinical signs, moved from BE to BK Day 2: BRSV diagnosed in BE Day 11: BRSV diagnosed in BK	One change T → C (synonymous) at position 9640, in gene L, unique to BK11J20. BK11J20 is the only field sample with > 1% diversity at this site (13 × C, 2 × T)	BE11J20 → BK11J20: 68%
Field outbreak: AI12U20, AI14U20, AJ11U20, AJ22U20, AL11U20	Day 0: AL received calves from AJ Day 11: Animal professional visited both herds Day 20 and 21: AI and AJ received visit from same organisation, respectively, for the same purpose, possibly same material. AI and AJ are geographically close (500–1000 m) Day 33: BRSV diagnosed in AI Day 34: BRSV diagnosed in AJ Day 36: BRSV diagnosed in AL	One change A → G (non-synonymous Ile → Met) at position 3981, in gene M, unique to AJ22U20. AJ22U20 is the only field sample with diversity > 1% at this site (319 × A, 409 × G). All other AI, AJ and AL consensus sequences were identical	*Between herd* No transmission links inferred with high confidence (max. probability 53%) *Within herd* AI12U20 → AI14U20: 53% AJ22U20 → AJ11U20: 51%

## Discussion

BRSV is a major cause of respiratory disease in cattle and the prevention of BRSV-infections is essential in most production systems. Many farmers rely on biosecurity measures rather than vaccination to stop the introduction of this virus into their herds when an upsurge of outbreaks occurs. To be efficient, biosecurity measures need to be tailored to the life cycle of the virus and to all the modes of virus transmission. With the final goal to understand how BRSV is introduced or maintained in farms, we assessed if analysis of full virus genome sequences could give better resolution than partial sequencing, to better trace the virus. For this purpose, in this first whole-genome sequencing study of BRSV, we have established a reliable deep-sequencing methodology using the Illumina MiSeq platform (objective 1) which we have used to quantify genomic diversity at the consensus and sub-consensus levels among experimental transmissions and among field outbreaks in Swedish cattle herds (objective 2). In addition to establishing a pipeline for whole-genome analysis of BRSV samples, we assessed the feasibility of using both consensus-level and sub-consensus-level genomic diversity to infer transmission links between individual cattle and between herds (objective 3). Finally, we investigated associations between sub-consensus diversity and sample type, Ct value and genomic location (objective 4). We achieved these aims by analysis of 47 whole viral genomes sequenced from experimental and field outbreak BRSV samples.

Analysis of variation between consensus sequences from three farms in Uppland and two in Jämtland showed strong partitioning of genomic variation between two major clades corresponding to the two regions, which are approximately 500 km apart, with very little variation at the local (< 20 km) scale within regions. The strong regional clustering of genomic variation suggests that these two virus populations have been evolving independently over timescales much greater than the days or weeks that likely separate the within-region samples, with very limited movement of infected animals between regions. This raises the exciting possibility that consensus sequences could be used to identify the region of origin of a new BRSV outbreak. However, analysis of a much larger sample of sequences from across Sweden will be required to determine the pattern of geographic structuring of consensus sequence variation and its potential utility for tracing outbreaks between regions. Such an analysis, if it included farms at the < 20 km scale, could also reveal whether the very low levels of within-region between-farm consensus sequence variation we have observed—and which are not promising for the prospect of tracing transmission between local farms—are typical of Sweden as a whole. It is notable that the low levels of consensus sequence variation at the local between-farm scale (0.5–20 km) was similar to that observed between animals along short experimental transmission chains. For example, no differences arose between the consensus sequences from experiment G over 21 days and three transmission steps.

Based on a limited number of studies in which partial sequences from the G and SH genes were used, it appears that viruses with similar genetic sequences cluster in time and space [12, 46]. The genetic diversity of BRSV observed in several other field studies could have been the result of introductions of new lineages from other areas, rather than reflecting a rapid evolution of BRSV following a local spread [14, 47–57]. Indeed, as previously shown by Larsen et al. [13] and Deplanche et al*.* [58] the consensus sequence of the G gene is very stable during passages in calves and cell cultures. The clinical signs increased gradually when BRSV was passaged between successively calves 3 times in experiment G (data not shown). Consequently, we expected genetic variation, possibly in other parts of the genome than the gene G. Nonetheless, despite an increase of clinical expression between the consecutive passages in calves (data not shown), no consensus level variation arose over the three passages, while the degree of sub-consensus diversity detected in viruses that were sequenced at each passage was not greater than the low level introduced in the challenge virus (Figure 3).

Analysis of consensus sequences inferred from deep sequencing data discards information on within-sample genomic diversity, which can be informative about transmission links. We therefore applied two methods that exploit within-sample diversity to infer bottleneck size [42] and transmission routes (*BadTrIP*) [26] between experimental samples where the transmission tree was known, and field outbreak samples where information on epidemiological distance was available. Neither the bottleneck size estimation method nor transmission route inference using *BadTrIP* was informative about transmission links, suggesting that within-sample diversity might not be of value in inferring local transmission routes. However, there are caveats to generalising pessimistically from these results. In the bottleneck analysis, there were too few samples with sufficiently high coverage to draw conclusions about the relationship between bottleneck size and transmission tree distance, therefore it remains possible that a relationship might be found using a larger sample set. Similarly, the severe down-sampling of coverage required to make *BadTrIP* computationally feasible is likely to have been costly in terms of statistical power, particularly in experiment G given its low levels of diversity relative to the other experiments. Thus, although these results are not promising, it is possible that other methods for inferring transmission routes from within-sample sequence diversity (e.g. [25]) applied to a larger sample of BRSV cases would have greater success in inferring transmission routes, particularly when combined with epidemiological data.

It was notable that bottleneck size estimates broadly overlapped between experiment G (17–112) and the field outbreak samples (20–516), despite the presumed greater epidemiological separation between the field outbreak samples, which would predict lower bottleneck size. Transmission bottleneck sizes have not previously been estimated for BRSV either experimentally or in natural outbreaks. Using a different statistical method, Lau et al. [59] estimated a lower-bound bottleneck size of 25 from experimental infections of adults with HRSV, which is within the range of bottleneck sizes estimated in our study. Applying similar sequencing and statistical methodology to our study, Orton et al. [60] estimated narrow FMDV transmission bottlenecks among outbreak samples (range 2–9) and among experimental transmissions (range 4–39). The implications for viral evolution (for example, in response to vaccination) of the moderate-to-narrow transmission bottlenecks estimated here are difficult to predict [61]. Narrow bottlenecks could in theory retard adaptation, for example, by increasing the frequency of deleterious mutations or reducing overall genetic variation, or facilitate it, by rapid establishment of inter-host viral diversity.

Levels of between- and within-sample diversity of G gene sequences from BRSV samples obtained from linked in vitro (*n* = 4) and in vivo (*n* = 3) experiments were assessed by Deplanche et al. [58]. No consensus-level changes were observed between the seven samples, consistent with the present study, while low but significant levels of sub-consensus diversity were demonstrated by sequencing 26–29 clones from each sample, estimated at 0.7–1.0 × 10^−3^ substitutions per nucleotide. We estimated using the binomial cumulative distribution function that a substitution rate of 10^–3^ per nucleotide operating uniformly across the whole genome would generate no diversity above the 1% error threshold used in this study, if we scaled up from Deplanche et al. (~27 sequences of ~660 nt in gene G) to our study (mean coverage of 1413 × of 14 562 nt across the whole genome). Although we should be cautious when comparing diversity between whole and partial genome sequences obtained using different sequencing technologies, the comparison illustrates that the significant levels of variation observed by Deplanche et al. are not necessarily higher than those observed across the whole genome in this study, where the least diverse sample showed 18 sites with diversity above the 1% error threshold. For other viruses, such as FMD, the degree of genetic diversity within a sample has previously been estimated to be 0.31 × 10^−3^ substitutions per nucleotide [21], 0.21 × 10^−3^ substitutions per nucleotide [62], and 0.07–0.77 × 10^−3^ substitutions per nucleotide [63] indicating similar short-term substitution rates largely consistent with the current study. The lack of genetic diversity within the BRSV samples and the strong stability of the consensus sequence could be explained by a stronger negative selection pressure on the vast majority of mutants arising following viral replication.

G and SH have been considered as the most variable parts of the genome and have therefore been used to study RSV diversity and evolution [12, 46]. Therefore, a secondary aim of this study was to compare partial genome sequences, specifically the more diverse SH + G region of the genome, with the whole genome in terms of the information available for tracing transmission routes. We confirmed that within-sample diversity is concentrated in the SH + G region, as evidenced by spikes in the distribution of diversity (Figure 4) and by comparison of diversity estimates between SH + G and both gene F and the whole genome (Figure 5). At the consensus level, we showed that the SH + G region is approximately twice as diverse and yielded an almost identical phylogenetic tree when compared with the whole genome. This result raises the possibility that sequencing of SH + G could be more cost-effective than whole-genome sequencing, for example in a large-scale analysis establishing the geographic structure of BRSV across Sweden. However, low coverage sequences did not tend to have higher coverage in the SH + G region than across the whole genome, suggesting that focusing on SH + G is unlikely to lead to the recovery of low-coverage samples. However, using specific nested RT-PCR for amplifying and sequencing this region might be of great help to characterize BRSV isolates in samples with low levels of virus RNA.

A limitation of the whole-genome sequencing approach is that, for sequences to be usable they must have adequate coverage, and mean coverage varies widely (range 1 × to 8614 × in this study). Three of the 50 samples sequenced were not analysed, having mean coverage below the 20 × threshold we set for consensus sequences, and 30 sequences did not reach the 500 × threshold for within-sample diversity analysis. Therefore, it would be valuable to be able to predict high coverage samples, and use this information to optimise coverage, and to identify low coverage samples before wasting resources on sequencing. In this study, sample collection method strongly predicted mean coverage, with the relatively convenient and unintrusive nasal swab method yielding higher coverage than bronchoalveolar lavage, suggesting that nasal swab should be used for future sampling of BRSV cases. However, Ct value did not predict mean coverage and therefore does not appear to have potential for prioritising high-coverage samples. However, samples with very high Ct values had already been filtered out, so this result suggests that additional filtering would not be useful.

Analysis of whole-genome sequences of BRSV can discriminate circulating isolates in distant areas. However, following in vivo transmission studies and analyses of a limited number of field samples, a very low level of mutations and diversity was observed among isolates with close epidemiological links. It can therefore be concluded that even though whole-genome sequencing can improve resolution to distinguish isolates that are circulating, the virus does not show a sufficient degree of rapid evolution and diversity to allow highly reliable tracing at the herd-level without the support of epidemiological information.

## Supplementary Information

**Additional file 1.** **Tables and figures giving additional detail on the design and outcomes of experiment G, NCBI accession numbers, and additional details of analyses of coverage, reliability, error threshold selection, bottleneck size estimation, and probabilistic transmission route inference.**

**Additional file 2.** **The genomic distribution of depth of coverage is plotted for 47 whole viral genome sequences from experiments A, B, C, D, F and G (*n* = 40), and from the field outbreak samples (*n* = 7).**

**Additional file 3.** **Alignments of variable sites from consensus sequences from the three experiments (C, D and F) where consensus-level variation was observed, and from the outbreak samples (O).**

## Data Availability

The nucleotide sequence data generated by the current study are available in the NCBI SRA and Nucleotide data bases and are collected under BioProject PRJNA893434. NCBI accession numbers are provided in Additional File 1B. All other bioinformatics and data analysis scripts are available in the University of Glasgow Enlighten Research Data repository (https://doi.org/10.5525/gla.researchdata.1287).
