# Supplementary material for: Evaluating the potential of whole-genome sequencing for tracing transmission routes in experimental infections and natural outbreaks of bovine respiratory syncytial virus
Source: Vet Res. 2022 Dec 12;53:107. doi: 10.1186/s13567-022-01127-9 (PMC9746130; doi:10.1186/s13567-022-01127-9)
Supplement: Supplementary file 1 — Additional file 1. Tables and figures giving additional detail on the design and outcomes of experiment G, NCBI accession numbers, and additional details of analyses of coverage, reliability, error threshold selection, bottleneck size estimation, and probabilistic transmission route inference. [file 13567_2022_1127_MOESM1_ESM.doc]

**Additional file 1: Supplementary tables and figures**

**Additional file 1A. Characteristics of animals included in experiment G.**

| Calf ID | Breed^a^ | Sex | Pen | Challenge^b^ | Age^c^ | Date^d^ | Sub-experiment |
| --- | --- | --- | --- | --- | --- | --- | --- |
| 7960 | SRB | M | 1 | 620 | 4.1 | 01/05/2018 | 1 |
| 7965 | SH | M | 1^e^ | 620 | 3.1 | 01/05/2018 | 1 |
| 2035 | SRB | F | 2 | 620 | 3.9 | 01/05/2018 | 1 |
| 7962 | SH | M | 2^f^ | 620 | 3.6 | 01/05/2018 | 1 |
| 7970 | SRB | M | 3 | 7960 | 5.6 | 29/05/2018 | 2 |
| 7971 | SRB | M | 4 | 2035 | 5.4 | 29/05/2018 | 2 |
| 2073 | SH | F | 5 | 7971 | 7.3 | 08/08/2018 | 3 |
| 7992 | SRB | M | 5 | 7971 | 7.0 | 08/08/2018 | 3 |
| 2078 | SRB | F | 6 | 7971 | 5.3 | 08/08/2018 | 3 |
| 2081 | SH | F | 6 | 7971 | 4.0 | 08/08/2018 | 3 |

^a^SRB: Swedish Red and White breed; SH: Swedish Holstein.

^b^Challenge performed with virus obtained from indicated calf.

^c^Age in weeks at challenge.

^d^Date of challenge.

^e^Additionally companion animal to 7971, pen 4; did not shed detectable virus Exp2, D0-7.

^f^Additionally companion animal to 7970, pen 3; did not shed detectable virus Exp2, D0-6. On D7, 2.3 TCID50 eq was detected in nasal swab.

**Additional file 1B. NCBI accession numbers for the Illumina FastQ files and the FASTA consensus sequences from the 50 sequenced samples. Accession numbers are provided for the BioProject, BioSample, Sequence Read Archive (SRA; for FastQ files), and GenBank Nucleotide (for FASTA consensus sequences) databases. Consensus sequences were not produced for three samples where mean coverage was less than 20×.**

| Sample ID | BioProject | BioSample | SRA Study | SRA run | GenBank |
| --- | --- | --- | --- | --- | --- |
| A_BRSVDKp7 | PRJNA893434 | SAMN31423185 | SRP404162 | SRR22019764 | OP715679 |
| B_8863BALd6 | PRJNA893434 | SAMN31423187 | SRP404162 | SRR22019725 | OP715681 |
| B_8863NSd4 | PRJNA893434 | SAMN31423188 | SRP404162 | SRR22019746 | OP715682 |
| B_8863NSd6 | PRJNA893434 | SAMN31423189 | SRP404162 | SRR22019735 | OP715683 |
| B_8871BALd6 | PRJNA893434 | SAMN31423190 | SRP404162 | SRR22019734 | OP715684 |
| B_8871NSd6 | PRJNA893434 | SAMN31423191 | SRP404162 | SRR22019763 | OP715685 |
| B_BRSVDKp7 | PRJNA893434 | SAMN31423186 | SRP404162 | SRR22019752 | OP715680 |
| C_7088BALd7 | PRJNA893434 | SAMN31423193 | SRP404162 | SRR22019715 | OP715687 |
| C_7088NSd6 | PRJNA893434 | SAMN31423194 | SRP404162 | SRR22019762 | OP715688 |
| C_7098BALd7 | PRJNA893434 | SAMN31423195 | SRP404162 | SRR22019761 | OP715689 |
| C_7098NSd6 | PRJNA893434 | SAMN31423196 | SRP404162 | SRR22019760 | OP715690 |
| C_7098NSd7 | PRJNA893434 | SAMN31423197 | SRP404162 | SRR22019717 | OP715691 |
| C_BRSVSnook | PRJNA893434 | SAMN31423192 | SRP404162 | SRR22019716 | OP715686 |
| D_3982BALd6 | PRJNA893434 | SAMN31423199 | SRP404162 | SRR22019757 | OP715693 |
| D_3982NSd6 | PRJNA893434 | SAMN31423200 | SRP404162 | SRR22019756 | OP715694 |
| D_3996BALd7 | PRJNA893434 | SAMN31423201 | SRP404162 | SRR22019759 | OP715695 |
| D_BRSV3761p2 | PRJNA893434 | SAMN31423198 | SRP404162 | SRR22019758 | OP715692 |
| F_8514NSd4 | PRJNA893434 | SAMN31423204 | SRP404162 | SRR22019750 | OP715698 |
| F_8514NSd5 | PRJNA893434 | SAMN31423205 | SRP404162 | SRR22019754 | OP715699 |
| F_8514NSd6 | PRJNA893434 | SAMN31423206 | SRP404162 | SRR22019733 | OP715700 |
| F_8514NSd7 | PRJNA893434 | SAMN31423203 | SRP404162 | SRR22019751 | OP715697 |
| F_8516NSd4 | PRJNA893434 | SAMN31423207 | SRP404162 | SRR22019732 | OP715701 |
| F_8516NSd5 | PRJNA893434 | SAMN31423208 | SRP404162 | SRR22019731 | OP715702 |
| F_8516NSd6 | PRJNA893434 | SAMN31423209 | SRP404162 | SRR22019730 | OP715703 |
| F_8516NSd7 | PRJNA893434 | SAMN31423210 | SRP404162 | SRR22019729 | OP715704 |
| F_8530NSd4 | PRJNA893434 | SAMN31423211 | SRP404162 | SRR22019728 | OP715705 |
| F_8530NSd5 | PRJNA893434 | SAMN31423212 | SRP404162 | SRR22019726 | OP715706 |
| F_8531BALd13 | PRJNA893434 | SAMN31423213 | SRP404162 | SRR22019727 | - |
| F_8531BALd5 | PRJNA893434 | SAMN31423214 | SRP404162 | SRR22019723 | OP715707 |
| F_8537BALd6 | PRJNA893434 | SAMN31423215 | SRP404162 | SRR22019724 | - |
| F_8537NSd4 | PRJNA893434 | SAMN31423216 | SRP404162 | SRR22019722 | OP715708 |
| F_8537NSd5 | PRJNA893434 | SAMN31423217 | SRP404162 | SRR22019755 | OP715709 |
| F_SnookBAL2018 | PRJNA893434 | SAMN31423202 | SRP404162 | SRR22019753 | OP715696 |
| G_2035BALd7 | PRJNA893434 | SAMN31423219 | SRP404162 | SRR22019719 | OP715711 |
| G_2073BALd7 | PRJNA893434 | SAMN31423220 | SRP404162 | SRR22019718 | OP715712 |
| G_2078BALd7 | PRJNA893434 | SAMN31423221 | SRP404162 | SRR22019749 | OP715713 |
| G_2081BALd7 | PRJNA893434 | SAMN31423222 | SRP404162 | SRR22019748 | OP715714 |
| G_7960BALd7 | PRJNA893434 | SAMN31423223 | SRP404162 | SRR22019747 | OP715715 |
| G_7970BALd7 | PRJNA893434 | SAMN31423224 | SRP404162 | SRR22019745 | OP715716 |
| G_7971BALd7 | PRJNA893434 | SAMN31423225 | SRP404162 | SRR22019744 | OP715717 |
| G_7992BALd7 | PRJNA893434 | SAMN31423226 | SRP404162 | SRR22019721 | OP715718 |
| G_BRSVSweden620p4 | PRJNA893434 | SAMN31423218 | SRP404162 | SRR22019720 | OP715710 |
| O_AI12U20 | PRJNA893434 | SAMN31423227 | SRP404162 | SRR22019743 | OP715719 |
| O_AI14U20 | PRJNA893434 | SAMN31423228 | SRP404162 | SRR22019742 | OP715720 |
| O_AJ11U20 | PRJNA893434 | SAMN31423229 | SRP404162 | SRR22019741 | OP715721 |
| O_AJ22U20 | PRJNA893434 | SAMN31423230 | SRP404162 | SRR22019740 | OP715722 |
| O_AJ25U20 | PRJNA893434 | SAMN31423231 | SRP404162 | SRR22019739 | - |
| O_AL11U20 | PRJNA893434 | SAMN31423232 | SRP404162 | SRR22019738 | OP715723 |
| O_BE11J20 | PRJNA893434 | SAMN31423233 | SRP404162 | SRR22019737 | OP715724 |
| O_BK11J20 | PRJNA893434 | SAMN31423234 | SRP404162 | SRR22019736 | OP715725 |

**Additional file 1C.** Estimated bottleneck sizes and 95% confidence intervals (CI) among the samples from experiment G (*n* = 6) and the field outbreak (*n* = 4) that passed the 500× coverage threshold (see main text, Figure 6). Because the direction of transmission was not known for the outbreak samples, bottleneck size was estimated in both directions. Distance for experiment G is the number of transmission steps separating the samples, while for the outbreak samples distance is an ordinal “epidemiological distance” scale derived from spatial and temporal proximity and contact tracing (see “Materials and methods” in main text for details).

| Sample source | Donor | Recipient | Distance | Bottleneck size estimate (95% CI) |
| --- | --- | --- | --- | --- |
| Experiment G | BRSVSweden620p4 | 2073BALd7 | 3 | 20 (13, 31) |
|  | 7971BALd7 | 2073BALd7 | 1 | 71 (35, 168) |
|  | BRSVSweden620p4 | 2078BALd7 | 3 | 32 (20, 49) |
|  | 7971BALd7 | 2078BALd7 | 1 | 81 (44, 152) |
|  | BRSVSweden620p4 | 7960BALd7 | 1 | 22 (14, 33) |
|  | BRSVSweden620p4 | 7971BALd7 | 2 | 17 (11, 26) |
|  | BRSVSweden620p4 | 7992BALd7 | 3 | 26 (16, 41) |
|  | 7971BALd7 | 7992BALd7 | 1 | 112 (51, 261) |
| Outbreak (forward) | AI14U20 | AJ11U20 | 3 | 399 (235, 657) |
|  | AI14U20 | AJ22U20 | 3 | 261 (130, 555) |
|  | AJ11U20 | AJ22U20 | 1 | 28 (18, 44) |
|  | AI14U20 | AL11U20 | 4 | 516 (319, 809) |
|  | AJ11U20 | AL11U20 | 2 | 34 (22, 51) |
|  | AJ22U20 | AL11U20 | 2 | 24 (16, 35) |
| Outbreak (reverse) | AI14U20 | AJ11U20 | 3 | 50 (34, 70) |
|  | AI14U20 | AJ22U20 | 3 | 27 (18, 39) |
|  | AJ11U20 | AJ22U20 | 1 | 20 (13, 30) |
|  | AI14U20 | AL11U20 | 4 | 280 (181, 413) |
|  | AJ11U20 | AL11U20 | 2 | 153 (87, 255) |
|  | AJ22U20 | AL11U20 | 2 | 158 (84, 300) |


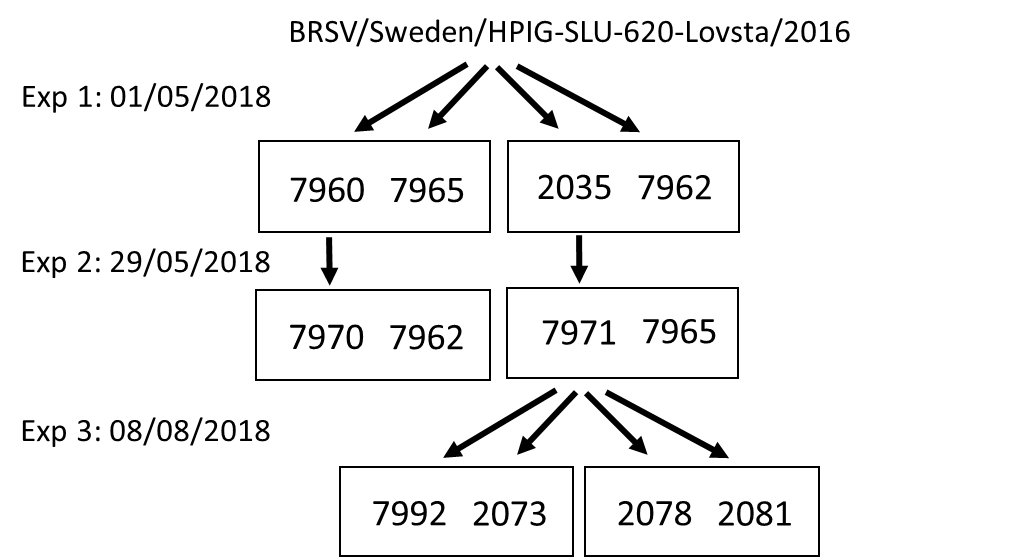


**Additional file 1D.** Time and source of challenge virus and allocation of calves to pens (boxes) in experiment G.

**
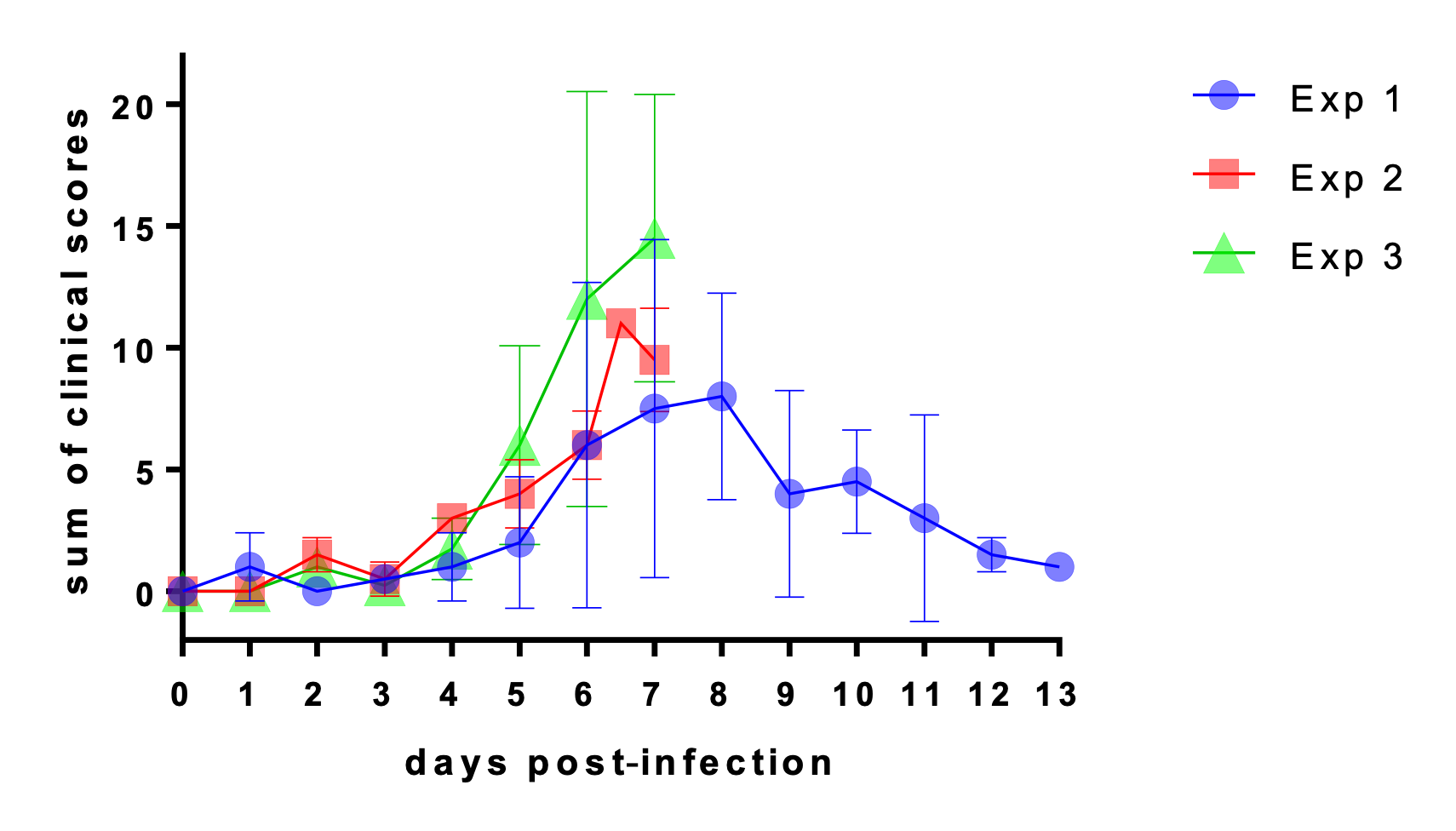
**

**Additional file 1E.** Sum of clinical scores of the ten calves in experiment G, performed during daily clinical examinations. The signs included one or several of the following clinical signs: mild cough, slight nasal discharge, increased respiratory rate, increased rectal temperature, wheezing on auscultation and abdominal dyspnea. None of the calves reached the humane endpoint (respiratory rate >100/min and severely depressed demeanour, lack of appetite for more than 24 h or rectal temperature >41 °C for more than 36 h).


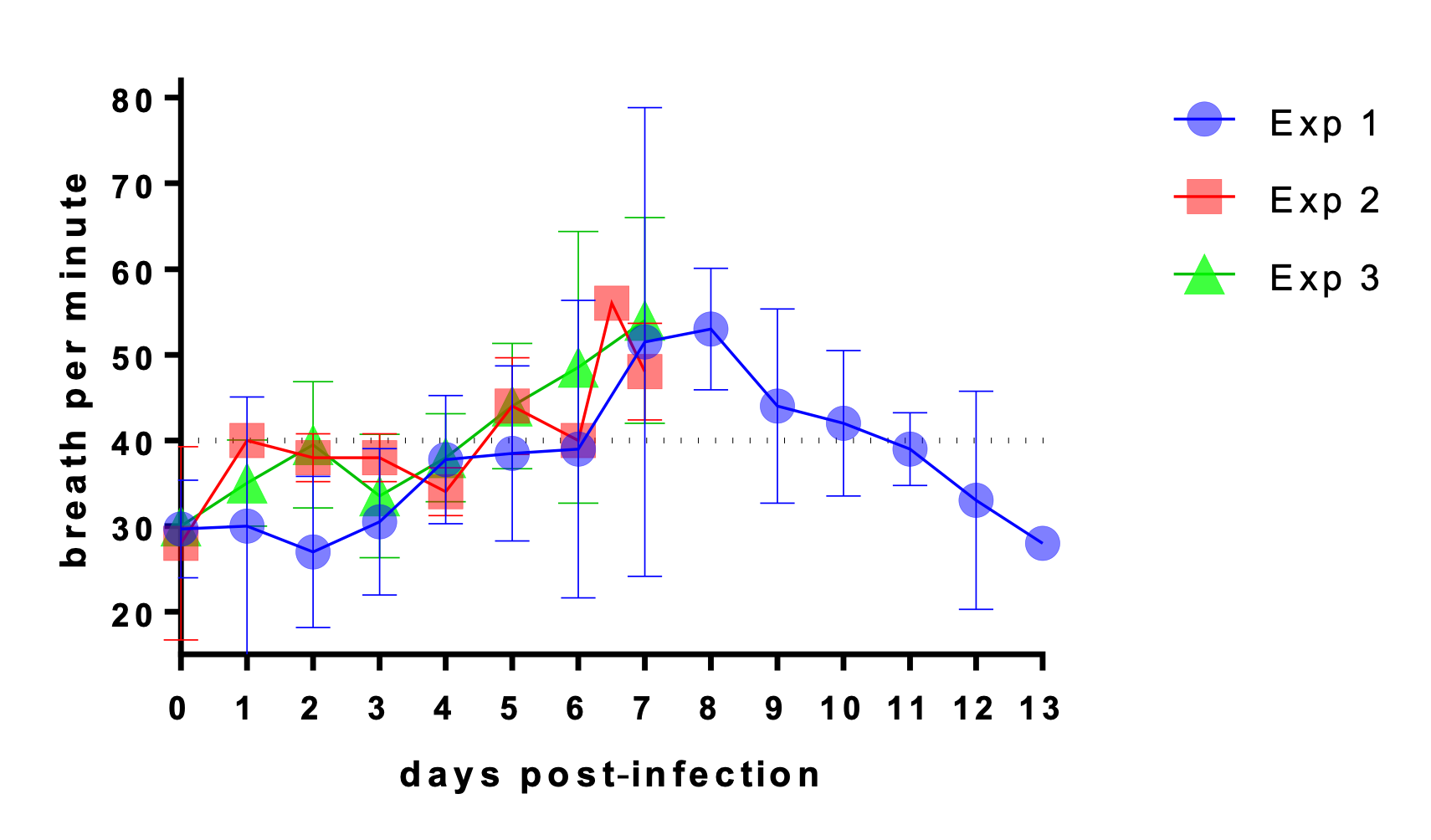


**Additional file 1F.** Respiratory rates of the ten calves in experiment G. Dotted line illustrate the upper limit of normal values in calves. Error bars represent standard deviation.

**
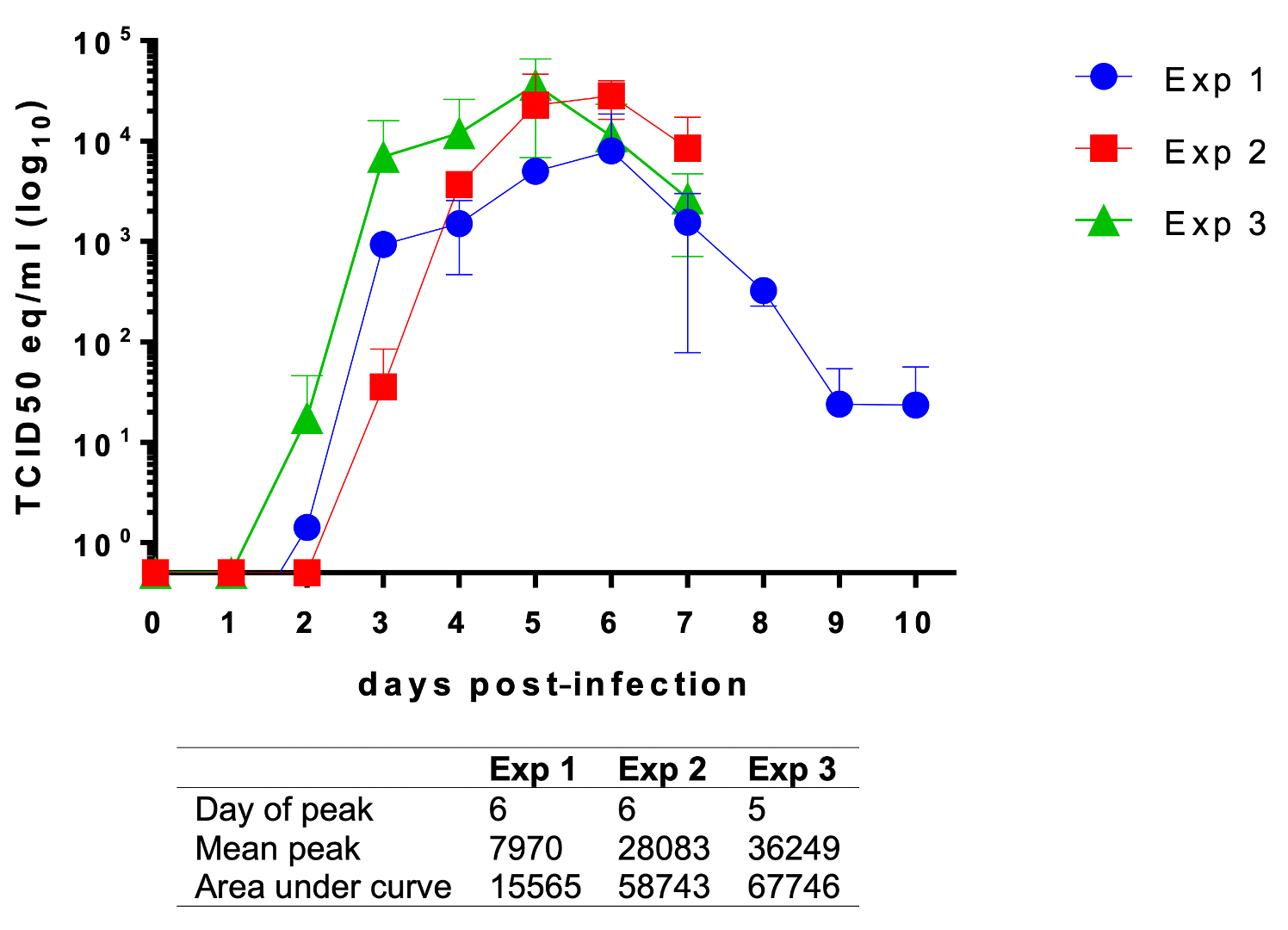
**

**Additional file 1G.** BRSV-RNA detected in daily nasal swabs of the ten calves in experiment G. The unit TCID_50_ equivalent (TCID_50_ eq.) was used since the standard curve used in the assay was based on a BRSV-infected cell lysate with a known titre. Error bars represent standard deviation.

**Additional file 1H.** Mean coverage compared between bronchoalveolar lavage (BAL) and nasal swab (NS) sampling methods.

**Additional file 1I** Reliability of NGS mismatch frequencies, gauged by the intraclass correlation coefficient (ICC), across repeated sequencing of 10 samples, calculated at error thresholds ranging from 0.1% to 2.0%. Each ICC was calculated after excluding sites with mismatch frequencies below the given error threshold. ICCs calculated from three permuted data sets (where ICC is expected to be zero) are also presented.


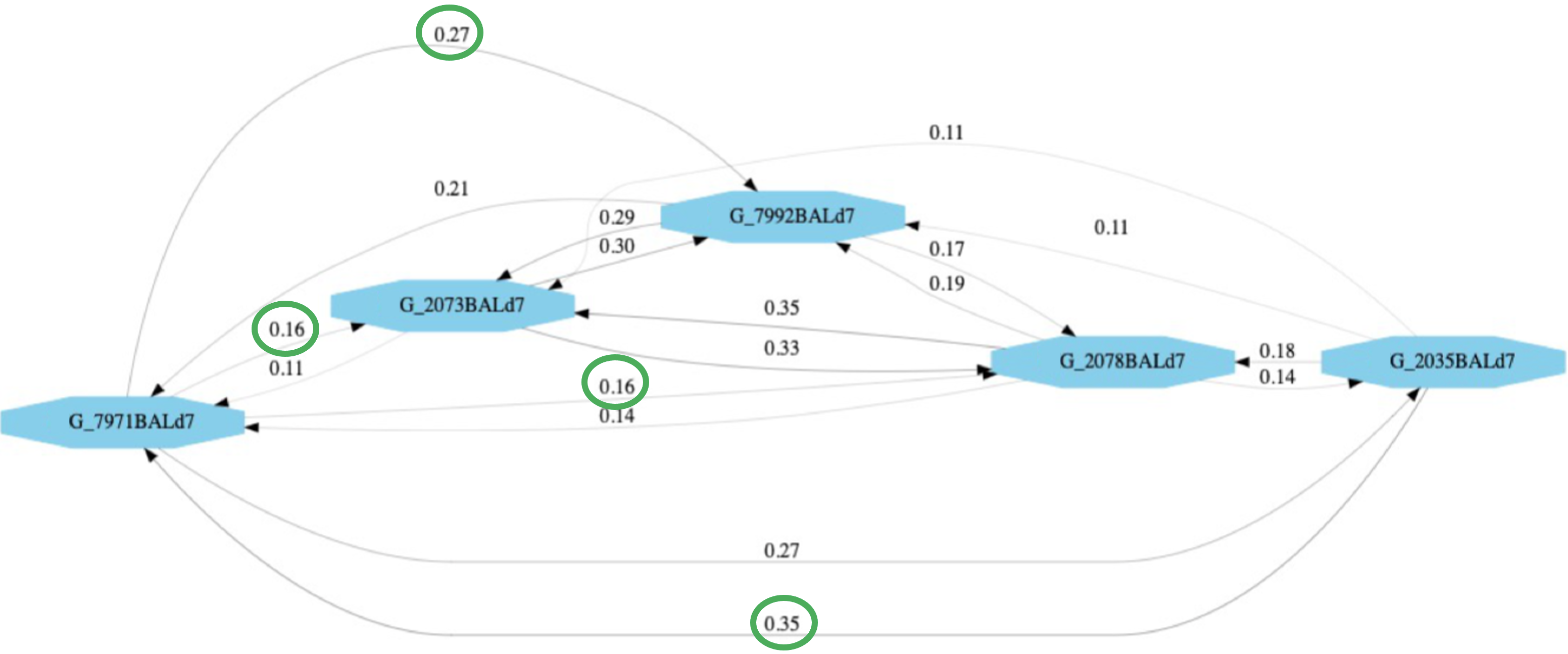


**Additional file 1J.** Probabilistic reconstruction using the *BadTrIP* method of transmission routes from experiment G, where the true transmission tree was known. Each blue octagon represents an animal, while arrows represent potential transmission routes. Estimated probabilities of transmission routes are given next to each arrow. The probabilities estimated for the true transmission routes are circled in green.


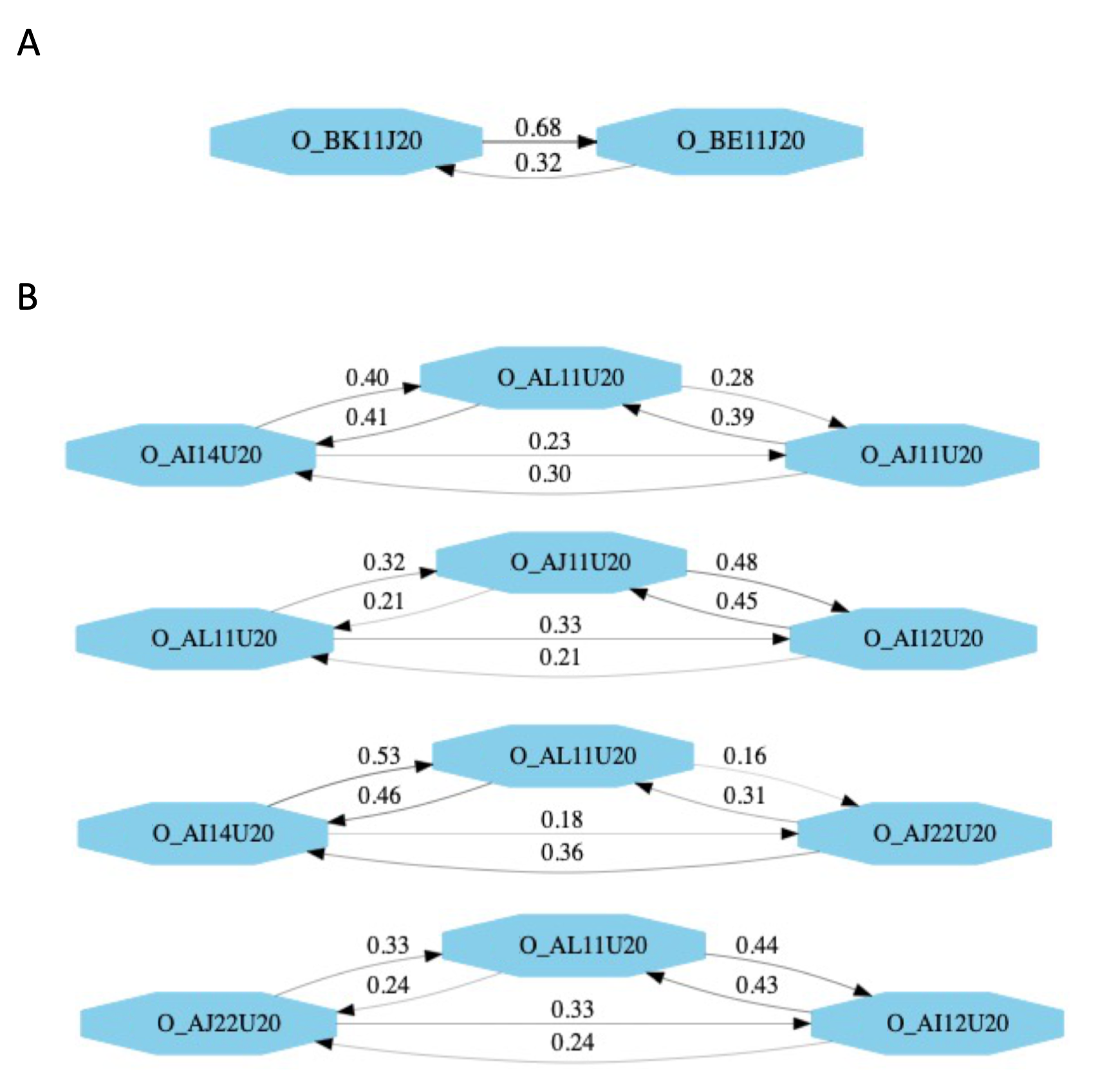


**Additional file 1K.** Probabilistic reconstruction of transmission links using *BadTrIP* among BRSV-infected farms linked by contact tracing (see main text Table 4 for details): farms BE and BK (A); and farms AI, AJ and AL (B). Each blue octagon represents an animal, while arrows represent potential transmission routes. Estimated probabilities of transmission routes are given next to each arrow. Four sample sets were run for farms AI, AJ and AL because two infected animals were available from each of AI and AJ, and not all could be analysed simultaneously due to computational intensity.
