# Supplementary material for: Evaluating the potential of whole-genome sequencing for tracing transmission routes in experimental infections and natural outbreaks of bovine respiratory syncytial virus
Source: Vet Res. 2022 Dec 12;53:107. doi: 10.1186/s13567-022-01127-9 (PMC9746130; doi:10.1186/s13567-022-01127-9)
Supplement: Supplementary file 2 — Additional file 2. The genomic distribution of depth of coverage is plotted for 47 whole viral genome sequences from experiments A, B, C, D, F and G (n = 40), and from the field outbreak samples (n = 7). [file 13567_2022_1127_MOESM2_ESM.pdf]

Additional File 2

The genomic distribution of depth of coverage is plotted for 47 whole viral genome sequences from experiments A, B, C, D, F and G (n = 40), and from the field outbreak samples (n = 7). The proportion of reads that mapped to the reference sequence is also given on each plot. Due to the omission of positions 1-578 from sequencing (see Methods), sequence coverage begins at position 579, at the 5' end of gene NS2. The challenge virus from experiment G, BRSV/Sweden/HPIG-SLU-620-Lovsta/2016, is abbreviated to G_BRSVSweden620p4.

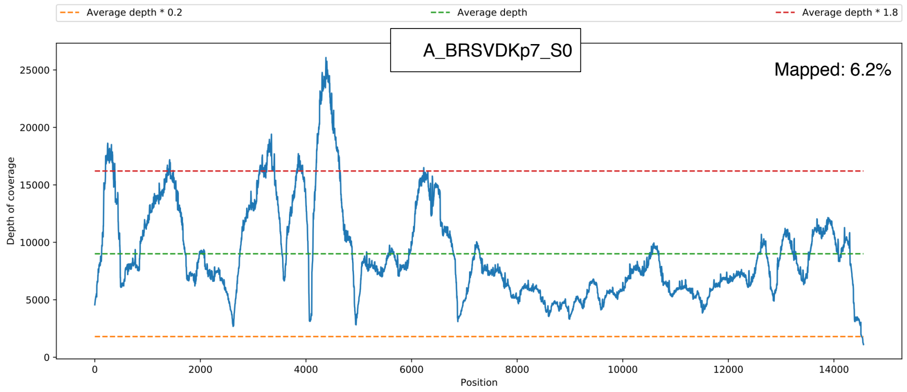

Average depth * 0.2
Average depth
Average depth * 1.8
A_BRSVDKp7_S0
Mapped: 6.2%
Depth of coverage
0
5000
10000
15000
20000
25000
Position
0
2000
4000
6000
8000
10000
12000
14000

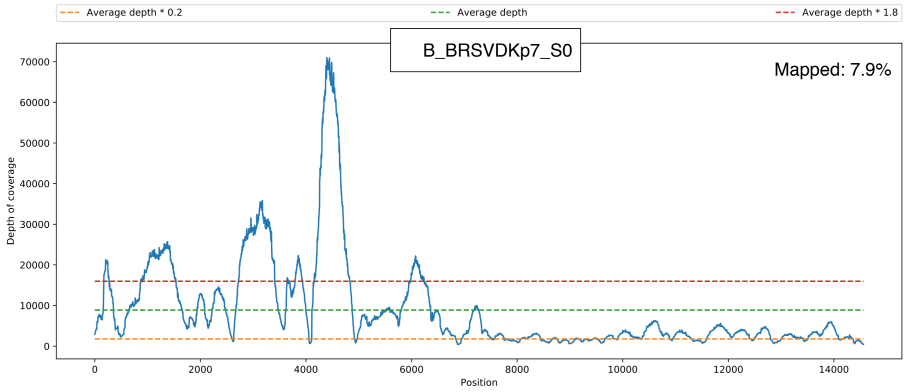
Average depth * 0.2
Average depth
Average depth * 1.8
B_BRSVDKp7_S0
Mapped: 7.9%
Depth of coverage
0
10000
20000
30000
40000
50000
60000
70000
0 2000 4000 6000 8000 10000 12000 14000
Position

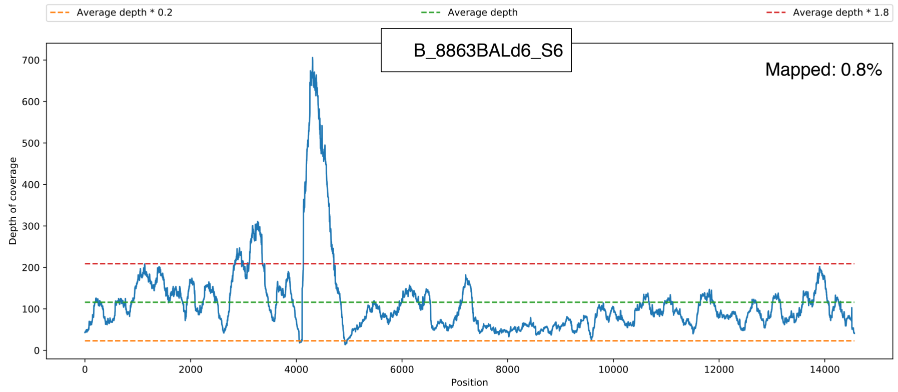
Average depth * 0.2
Average depth
Average depth * 1.8
B_8863BALd6_S6
Mapped: 0.8%
Depth of coverage
0
100
200
300
400
500
600
700
0 2000 4000 6000 8000 10000 12000 14000
Position

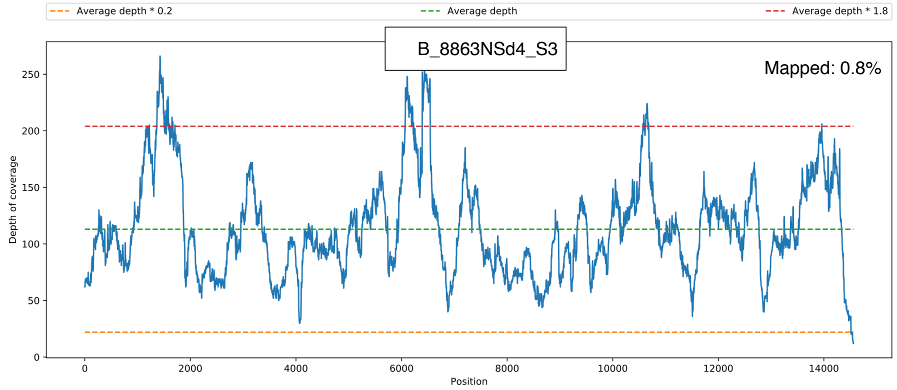
Average depth * 0.2
Average depth
Average depth * 1.8
B_8863NSd4_S3
Mapped: 0.8%
Depth of coverage
0
50
100
150
200
250
0 2000 4000 6000 8000 10000 12000 14000
Position

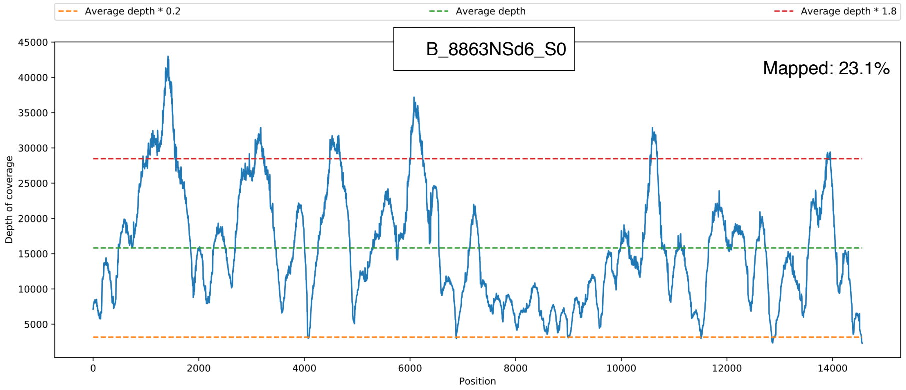
Average depth * 0.2
Average depth
Average depth * 1.8
B_8863NSd6_S0
Mapped: 23.1%
Depth of coverage
5000
10000
15000
20000
25000
30000
35000
40000
45000
0 2000 4000 6000 8000 10000 12000 14000
Position

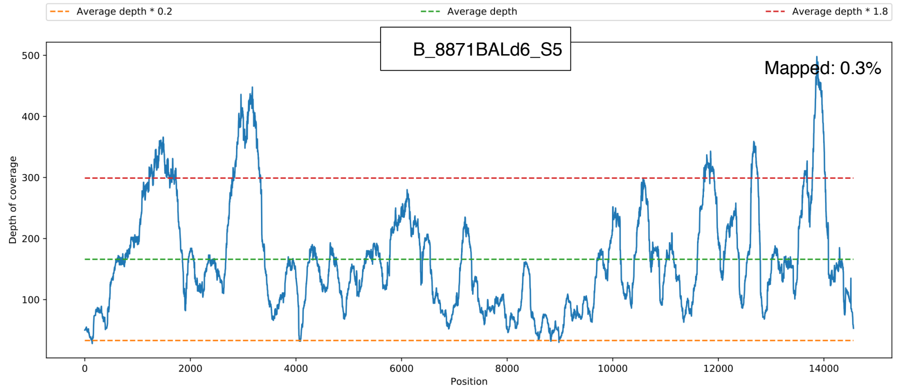
Average depth * 0.2
Average depth
Average depth * 1.8
B_8871BALd6_S5
Mapped: 0.3%
Depth of coverage
100
200
300
400
500
0 2000 4000 6000 8000 10000 12000 14000
Position

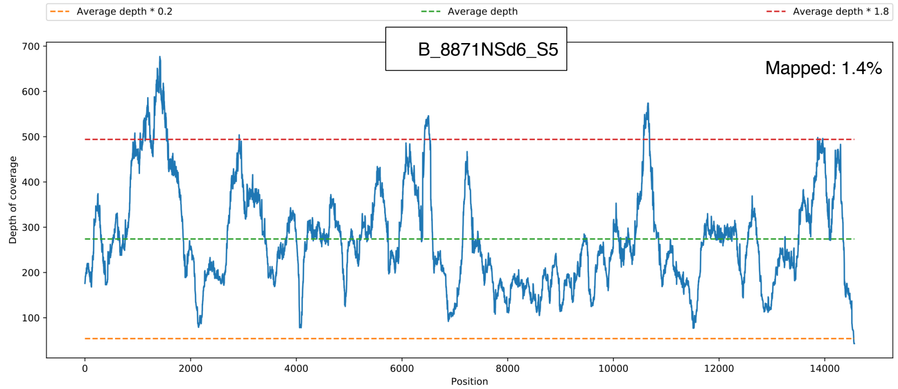
Average depth * 0.2
Average depth
Average depth * 1.8
B_8871NSd6_S5
Mapped: 1.4%
Depth of coverage
100
200
300
400
500
600
700
0 2000 4000 6000 8000 10000 12000 14000
Position

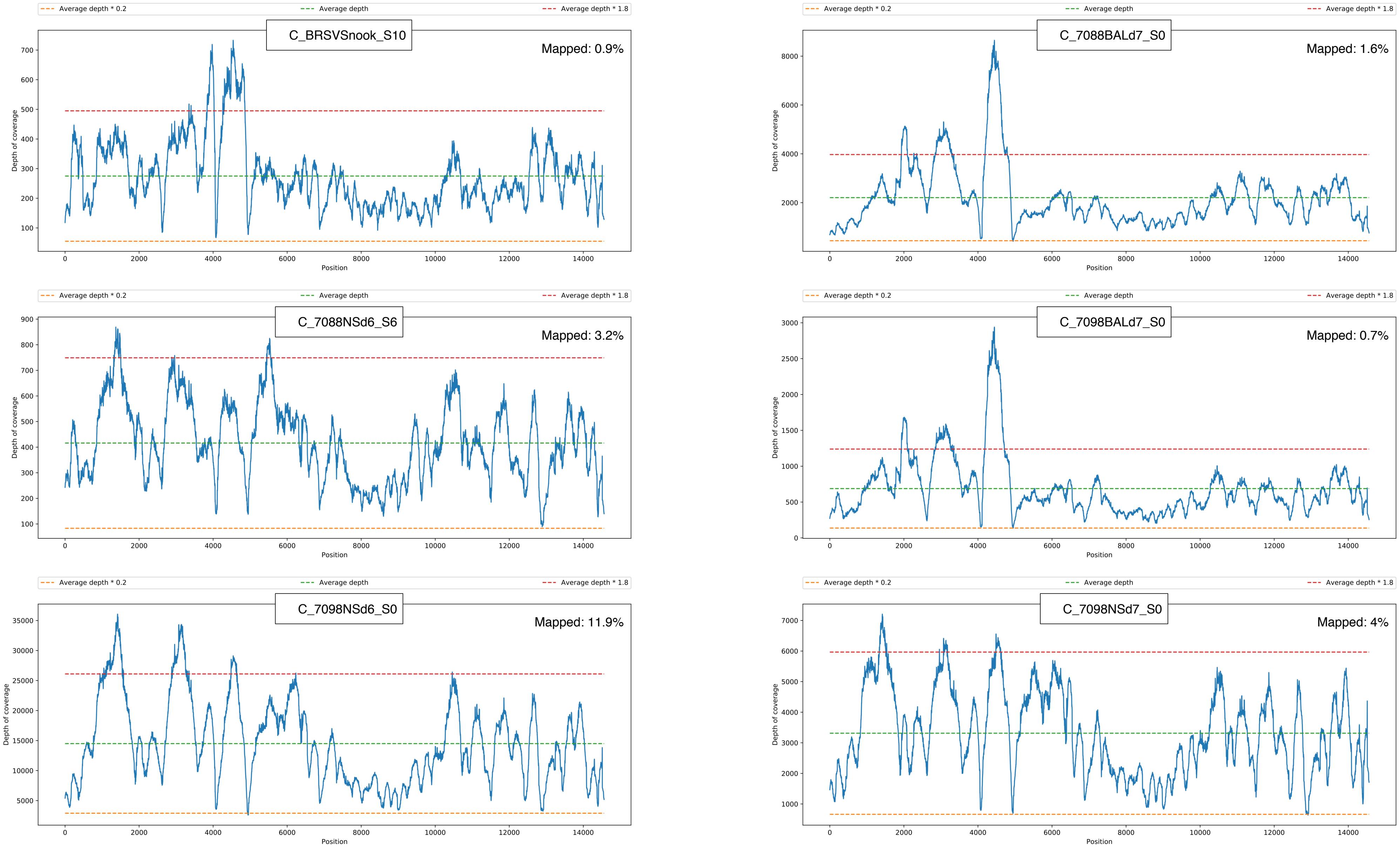
Average depth * 0.2
Average depth
Average depth * 1.8
C_BRSVSnook_S10
Mapped: 0.9%
C_7088BALd7_S0
Mapped: 1.6%
C_7088NSd6_S6
Mapped: 3.2%
C_7098BALd7_S0
Mapped: 0.7%
C_7098NSd6_S0
Mapped: 11.9%
C_7098NSd7_S0
Mapped: 4%
Depth of coverage
Position

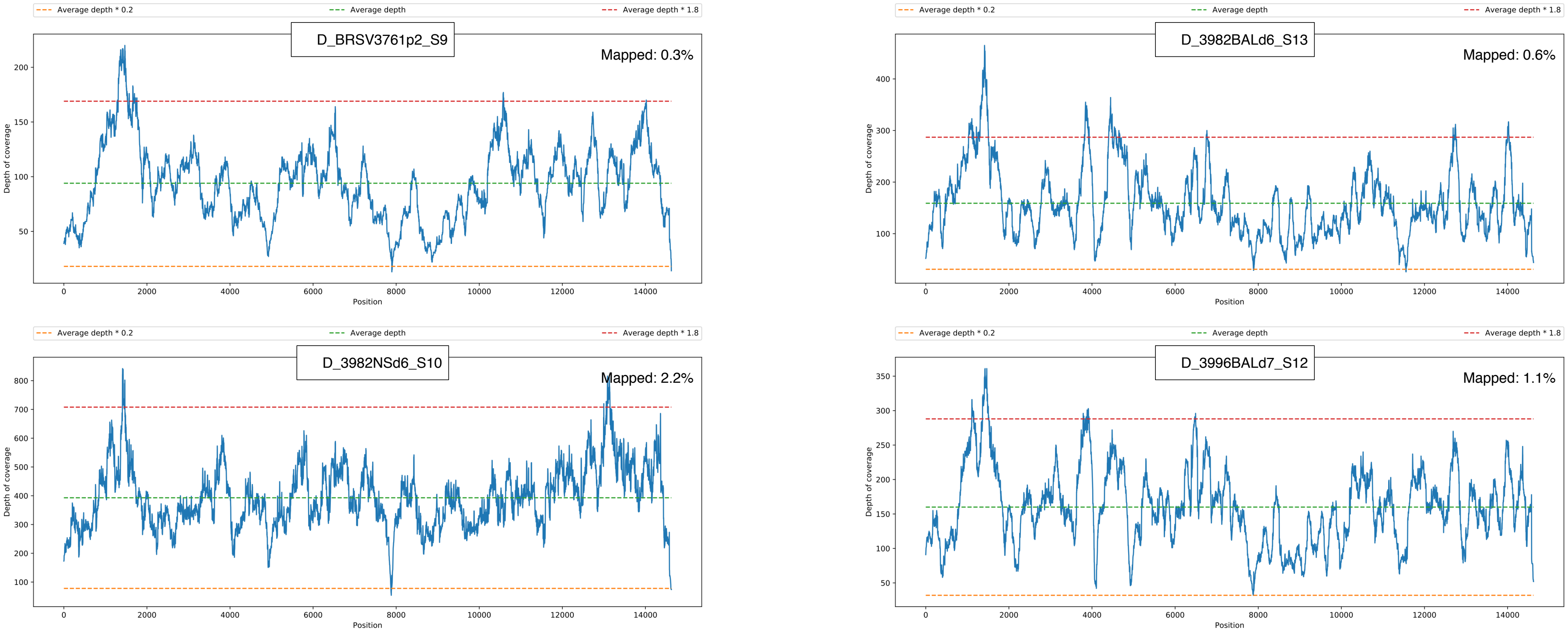
Average depth * 0.2
Average depth
Average depth * 1.8
D_BRSV3761p2_S9
Mapped: 0.3%
Depth of coverage
Position
D_3982BALd6_S13
Mapped: 0.6%
D_3982NSd6_S10
Mapped: 2.2%
D_3996BALd7_S12
Mapped: 1.1%
0
2000
4000
6000
8000
10000
12000
14000

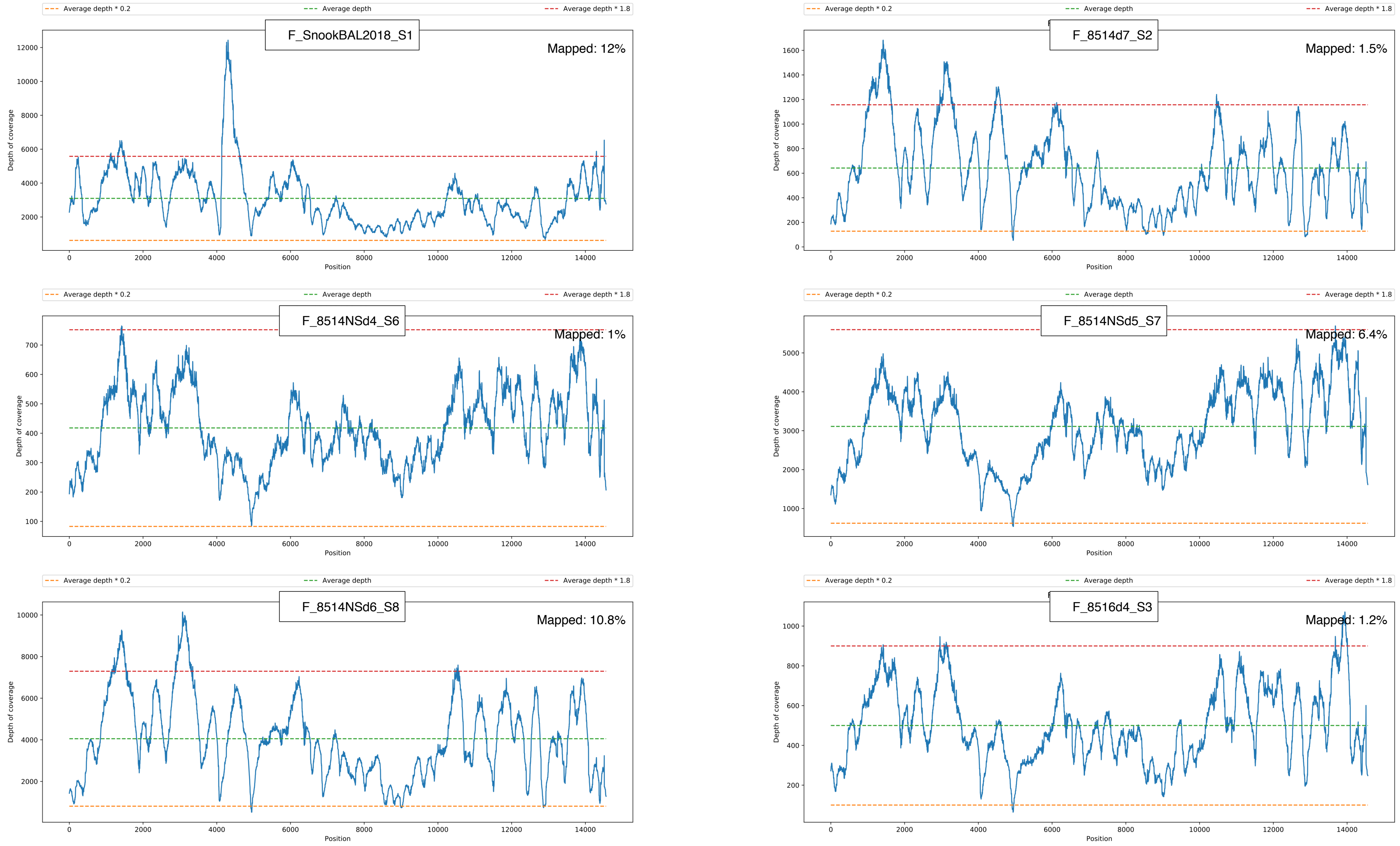

Average depth * 0.2
Average depth
Average depth * 1.8
F_SnookBAL2018_S1
Mapped: 12%
F_8514d7_S2
Mapped: 1.5%
F_8514NSd4_S6
Mapped: 1%
F_8514NSd5_S7
Mapped: 6.4%
F_8514NSd6_S8
Mapped: 10.8%
F_8516d4_S3
Mapped: 1.2%
Depth of coverage
Position

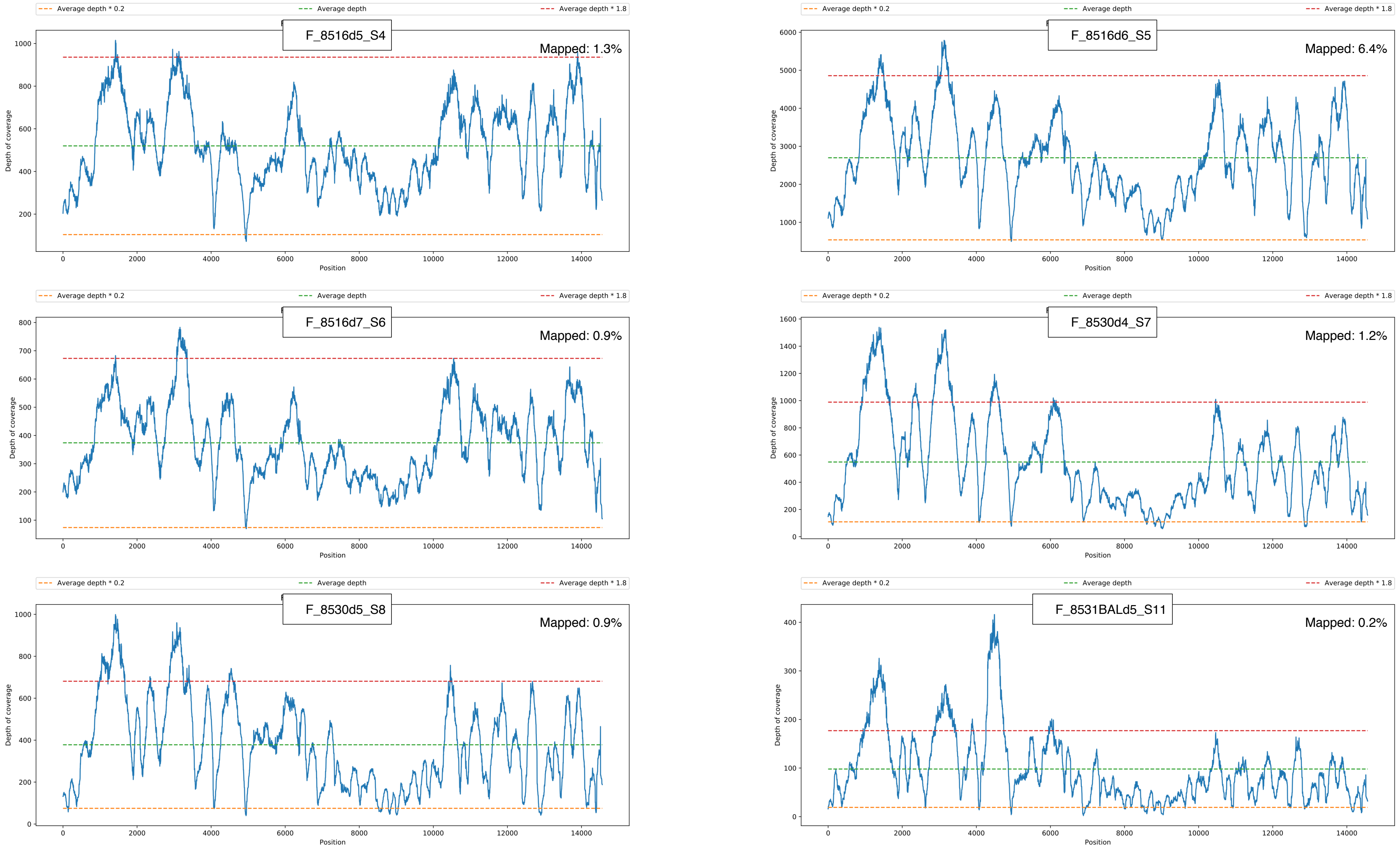
Average depth * 0.2
Average depth
Average depth * 1.8
F_8516d5_S4
Mapped: 1.3%
F_8516d6_S5
Mapped: 6.4%
F_8516d7_S6
Mapped: 0.9%
F_8530d4_S7
Mapped: 1.2%
F_8530d5_S8
Mapped: 0.9%
F_8531BALd5_S11
Mapped: 0.2%
Depth of coverage
Position

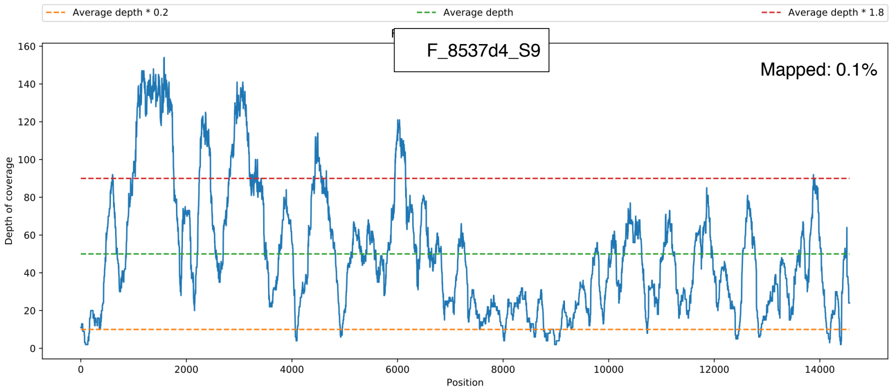
Average depth * 0.2
Average depth
Average depth * 1.8
F_8537d4_S9
Mapped: 0.1%
Depth of coverage
Position
0
20
40
60
80
100
120
140
160
0
2000
4000
6000
8000
10000
12000
14000

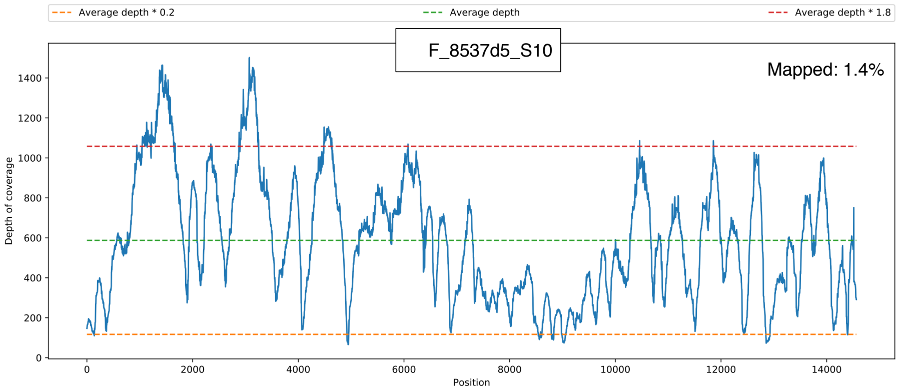
Average depth * 0.2
Average depth
Average depth * 1.8
F_8537d5_S10
Mapped: 1.4%
Depth of coverage
Position
0
200
400
600
800
1000
1200
1400
0
2000
4000
6000
8000
10000
12000
14000

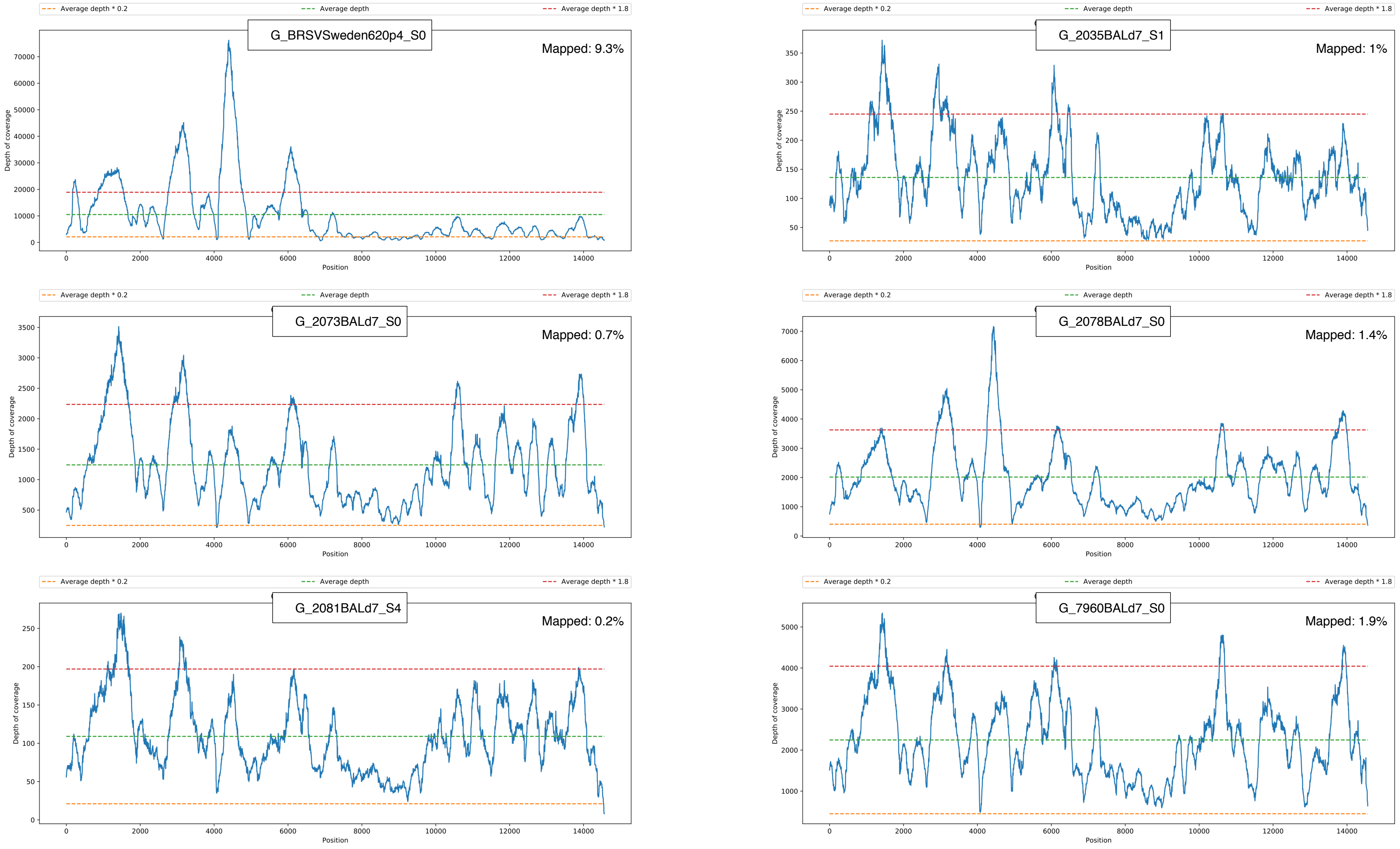
G_BRSVSweden620p4_S0
Mapped: 9.3%
G_2035BALd7_S1
Mapped: 1%
G_2073BALd7_S0
Mapped: 0.7%
G_2078BALd7_S0
Mapped: 1.4%
G_2081BALd7_S4
Mapped: 0.2%
G_7960BALd7_S0
Mapped: 1.9%
Average depth * 0.2
Average depth
Average depth * 1.8
Depth of coverage
Position

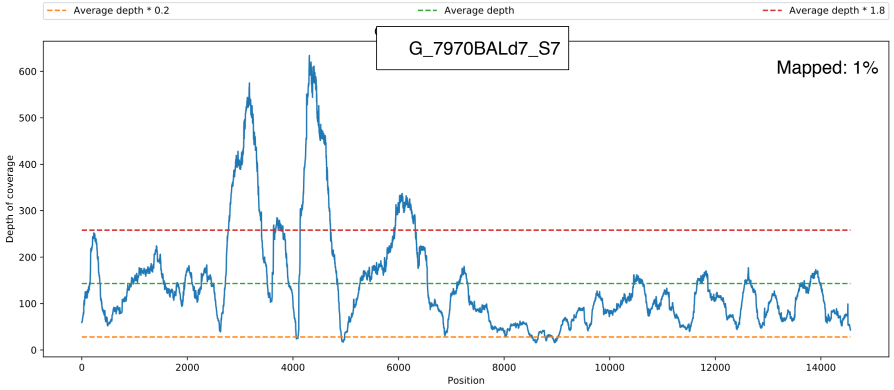
Average depth * 0.2
Average depth
Average depth * 1.8
G_7970BALd7_S7
Mapped: 1%
Depth of coverage
Position
0
100
200
300
400
500
600
0
2000
4000
6000
8000
10000
12000
14000

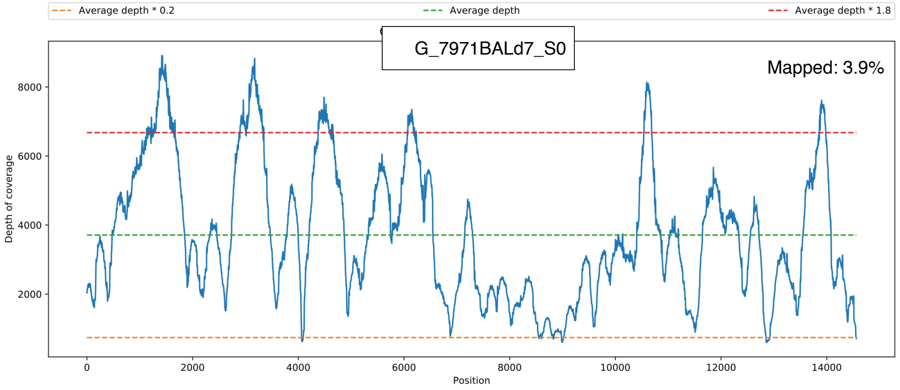
Average depth * 0.2
Average depth
Average depth * 1.8
G_7971BALd7_S0
Mapped: 3.9%
Depth of coverage
Position
2000
4000
6000
8000
0
2000
4000
6000
8000
10000
12000
14000

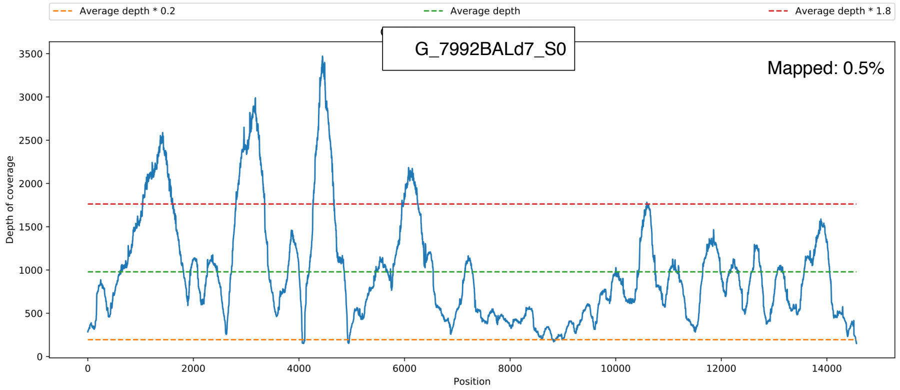
Average depth * 0.2
Average depth
Average depth * 1.8
G_7992BALd7_S0
Mapped: 0.5%
Depth of coverage
Position
0
500
1000
1500
2000
2500
3000
3500
0
2000
4000
6000
8000
10000
12000
14000

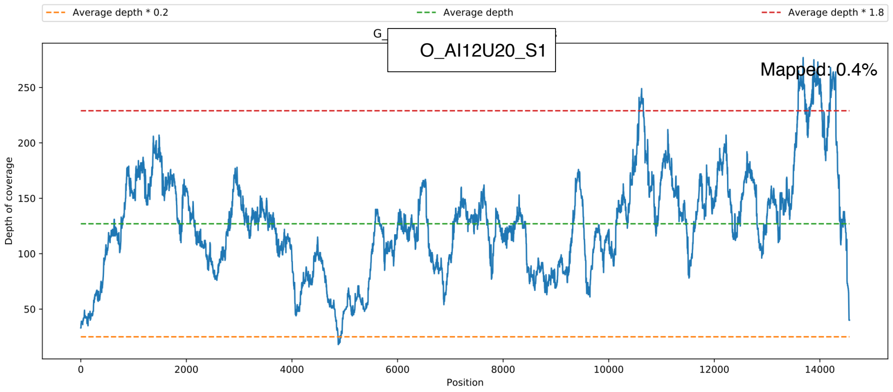
Average depth * 0.2
Average depth
Average depth * 1.8
G_
O_AI12U20_S1
Mapped: 0.4%
Depth of coverage
Position

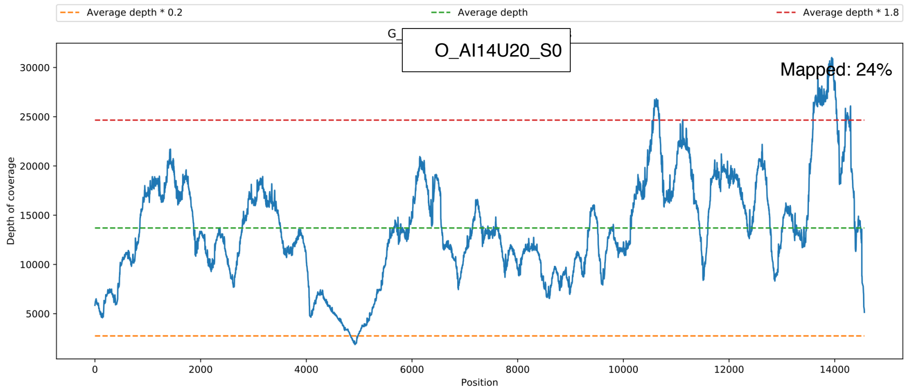
Average depth * 0.2
Average depth
Average depth * 1.8
G_
O_AI14U20_S0
Mapped: 24%
Depth of coverage
Position

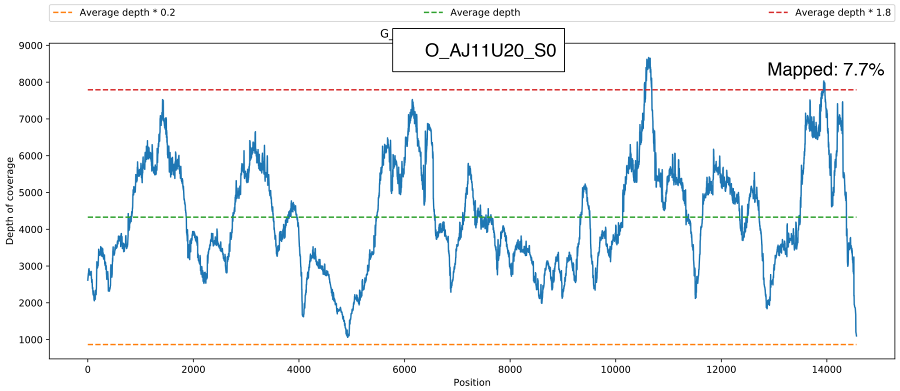
Average depth * 0.2
Average depth
Average depth * 1.8
G_
O_AJ11U20_S0
Mapped: 7.7%
Depth of coverage
Position

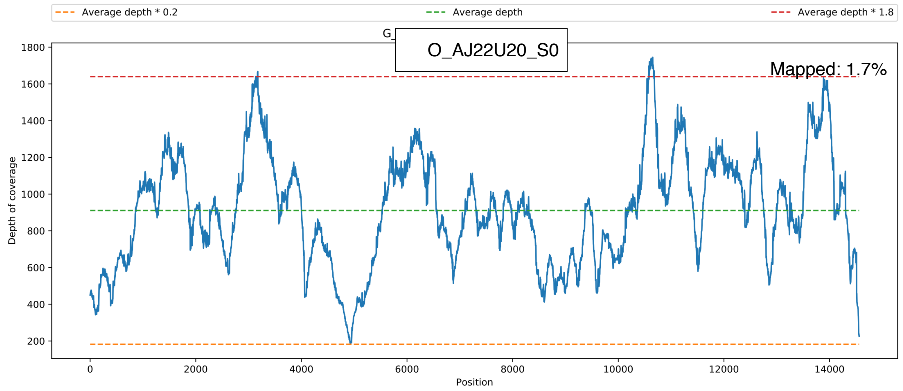
Average depth * 0.2
Average depth
Average depth * 1.8
G_
O_AJ22U20_S0
Mapped: 1.7%
Depth of coverage
Position

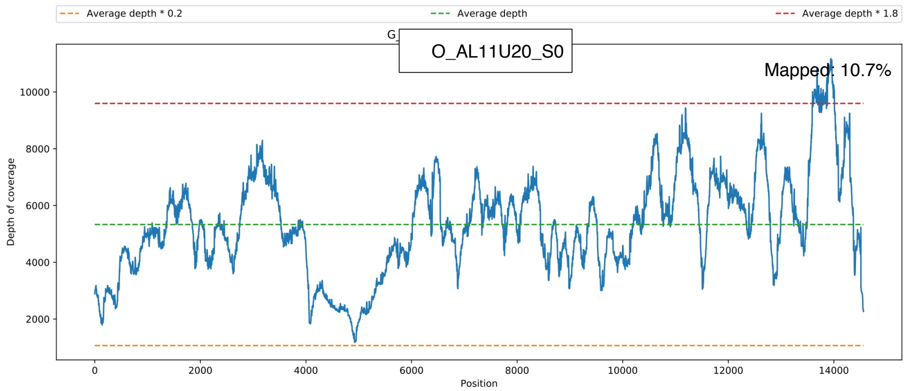
Average depth * 0.2
Average depth
Average depth * 1.8
G_
O_AL11U20_S0
Mapped: 10.7%
Depth of coverage
Position

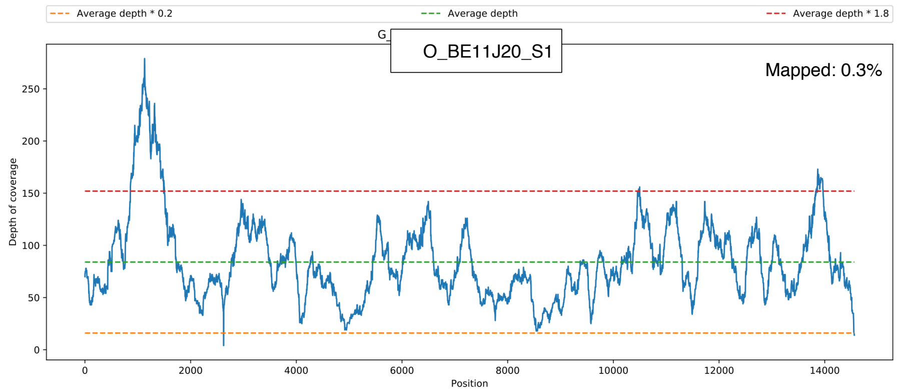
Average depth * 0.2
Average depth
Average depth * 1.8
G_
O_BE11J20_S1
Mapped: 0.3%
Depth of coverage
Position

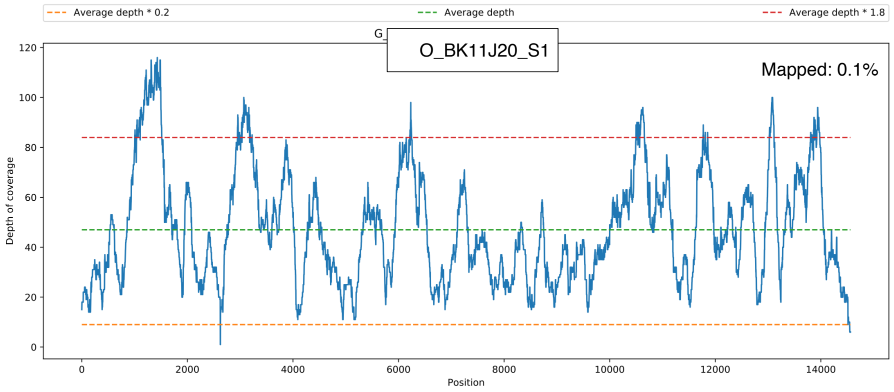
Average depth * 0.2
Average depth
Average depth * 1.8
G_
O_BK11J20_S1
Mapped: 0.1%
Depth of coverage
Position
0
20
40
60
80
100
120
0
2000
4000
6000
8000
10000
12000
14000
