# Supplementary material for: Evaluating the potential of whole-genome sequencing for tracing transmission routes in experimental infections and natural outbreaks of bovine respiratory syncytial virus
Source: Vet Res. 2022 Dec 12;53:107. doi: 10.1186/s13567-022-01127-9 (PMC9746130; doi:10.1186/s13567-022-01127-9)
Supplement: Supplementary file 3 — Additional file 3. Alignments of variable sites from consensus sequences from the three experiments (C, D and F) where consensus-level variation was observed, and from the outbreak samples (O). [file 13567_2022_1127_MOESM3_ESM.pdf]

Additional File 3

Alignments of variable sites from consensus sequences from the three experiments (C, D and F) where consensus-level variation was observed, and from the outbreak samples (O). Where space allowed, sequence flanking each variable site is shown. For each sample at each site, coverage is given, as is the percentage frequency of non-reference-sequence variants. For example, in experiment C there was a single variable site at position 8750 (note that because of the omission of the first 578 bases, this position is 9328 relative to NCBI Reference Sequence NC_038272.1; see main text Table 3). The depth of sequencing coverage at that position for the challenge virus, C_BRSVSnook, was 106. The consensus base was A, but there was a common minority variant at a frequency of 39.6%.

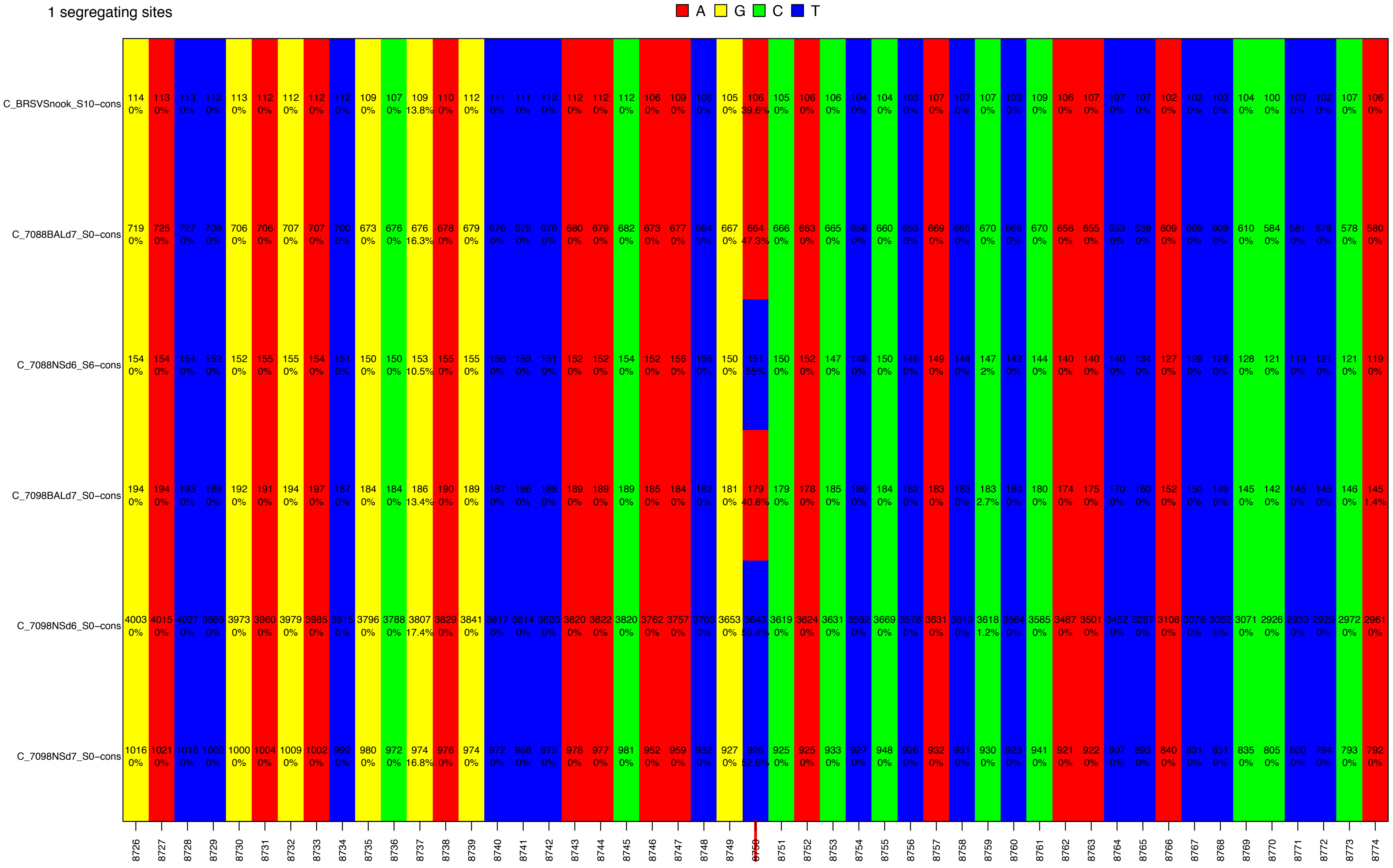
1 segregating sites
A
G
C
T
C_BRSVSnook_S10−cons
C_7088BALd7_S0−cons
C_7088NSd6_S6−cons
C_7098BALd7_S0−cons
C_7098NSd6_S0−cons
C_7098NSd7_S0−cons
8726 8727 8728 8729 8730 8731 8732 8733 8734 8735 8736 8737 8738 8739 8740 8741 8742 8743 8744 8745 8746 8747 8748 8749 8750 8751 8752 8753 8754 8755 8756 8757 8758 8759 8760 8761 8762 8763 8764 8765 8766 8767 8768 8769 8770 8771 8772 8773 8774

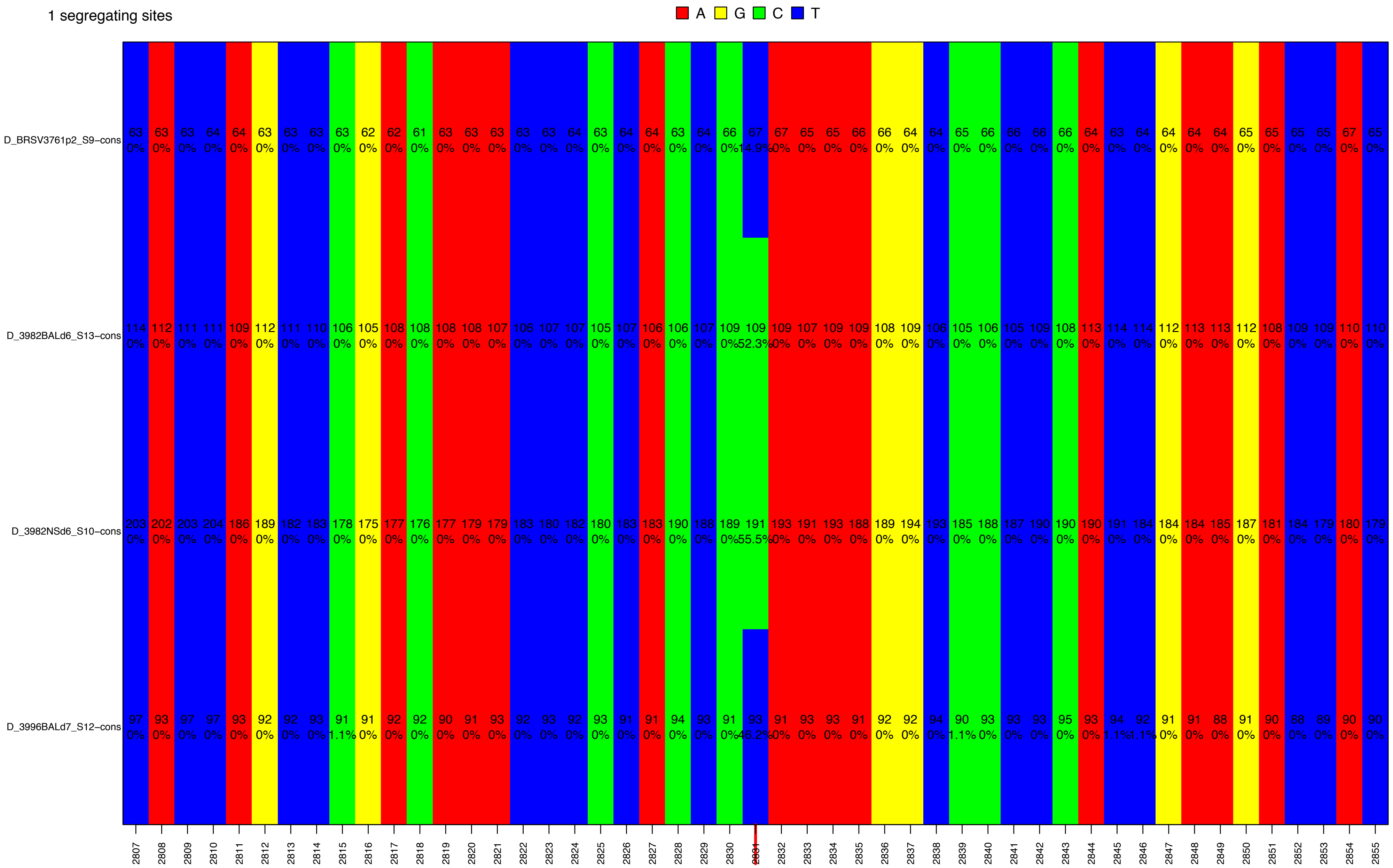

1 segregating sites
A G C T
D_BRSV3761p2_S9-cons
D_3982BALd6_S13-cons
D_3982NSd6_S10-cons
D_3996BALd7_S12-cons
63 63 63 64 64 63 63 63 63 62 62 61 63 63 63 63 63 64 63 64 64 63 64 66 67 67 65 65 66 66 64 64 65 66 66 66 66 64 63 64 64 64 64 65 65 65 65 67 65
0% 0% 0% 0% 0% 0% 0% 0% 0% 0% 0% 0% 0% 0% 0% 0% 0% 0% 0% 0% 0% 0% 0% 0% 14.9% 0% 0% 0% 0% 0% 0% 0% 0% 0% 0% 0% 0% 0% 0% 0% 0% 0% 0% 0% 0% 0% 0% 0% 0%
114 112 111 111 109 112 111 110 106 105 108 108 108 108 107 106 107 107 105 107 106 106 107 109 109 109 107 109 109 108 109 106 105 106 105 109 108 113 114 114 112 113 113 112 108 109 109 110 110
0% 0% 0% 0% 0% 0% 0% 0% 0% 0% 0% 0% 0% 0% 0% 0% 0% 0% 0% 0% 0% 0% 0% 0% 52.3% 0% 0% 0% 0% 0% 0% 0% 0% 0% 0% 0% 0% 0% 0% 0% 0% 0% 0% 0% 0% 0% 0% 0% 0%
203 202 203 204 186 189 182 183 178 175 177 176 177 179 179 183 180 182 180 183 183 190 188 189 191 193 191 193 188 189 194 193 185 188 187 190 190 190 191 184 184 184 185 187 181 184 179 180 179
0% 0% 0% 0% 0% 0% 0% 0% 0% 0% 0% 0% 0% 0% 0% 0% 0% 0% 0% 0% 0% 0% 0% 0% 55.5% 0% 0% 0% 0% 0% 0% 0% 0% 0% 0% 0% 0% 0% 0% 0% 0% 0% 0% 0% 0% 0% 0% 0% 0%
97 93 97 97 93 92 92 93 91 91 92 92 90 91 93 92 93 92 93 91 91 94 93 91 93 91 93 93 91 92 92 94 90 93 93 93 95 93 94 92 91 91 88 91 90 88 89 90 90
0% 0% 0% 0% 0% 0% 0% 0% 1.1% 0% 0% 0% 0% 0% 0% 0% 0% 0% 0% 0% 0% 0% 0% 0% 46.2% 0% 0% 0% 0% 0% 0% 0% 1.1% 0% 0% 0% 0% 0% 1.1% 1.1% 0% 0% 0% 0% 0% 0% 0% 0% 0%
2807 2808 2809 2810 2811 2812 2813 2814 2815 2816 2817 2818 2819 2820 2821 2822 2823 2824 2825 2826 2827 2828 2829 2830 2831 2832 2833 2834 2835 2836 2837 2838 2839 2840 2841 2842 2843 2844 2845 2846 2847 2848 2849 2850 2851 2852 2853 2854 2855

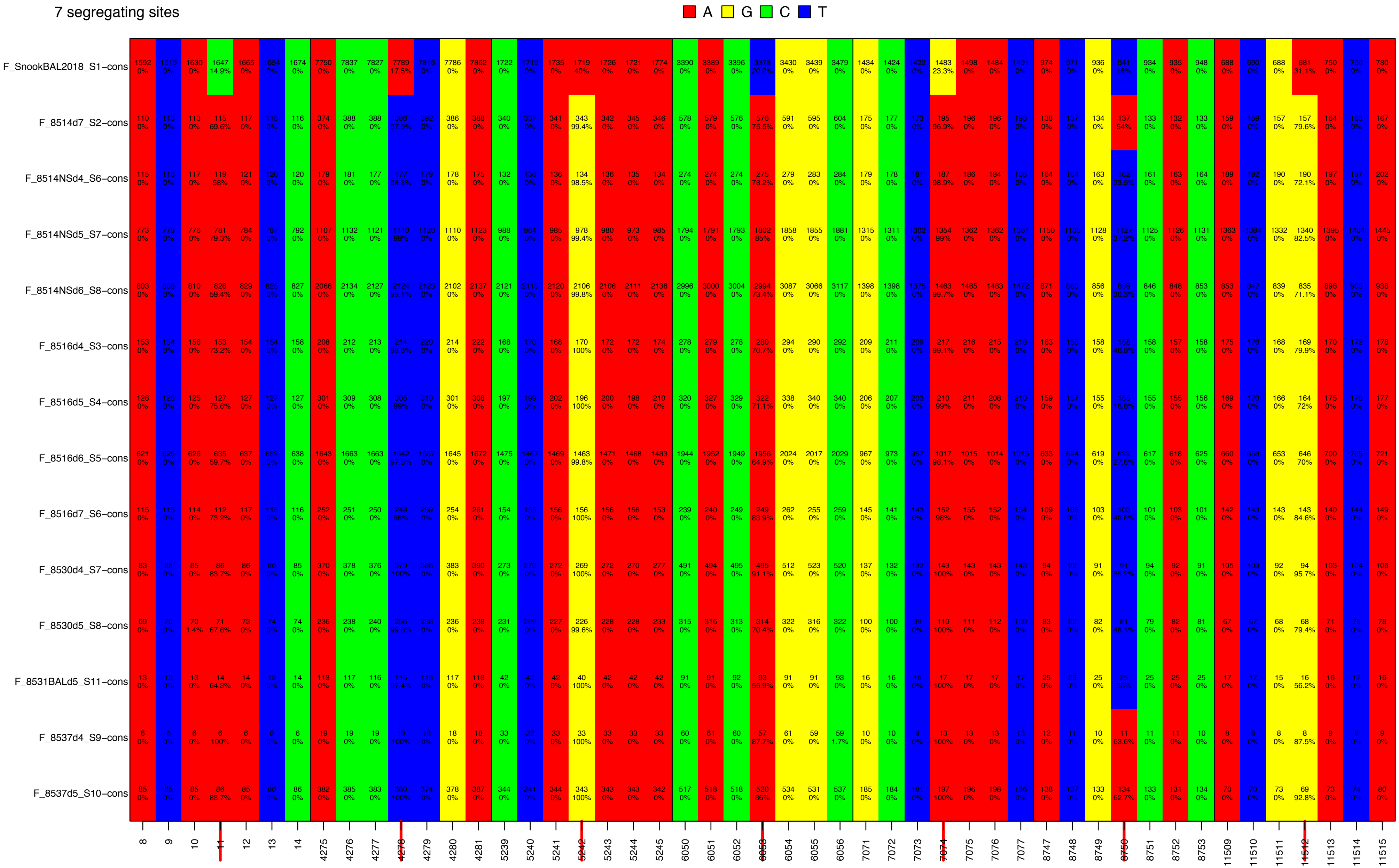
7 segregating sites
A G C T
F_SnookBAL2018_S1-cons
F_8514d7_S2-cons
F_8514NSd4_S6-cons
F_8514NSd5_S7-cons
F_8514NSd6_S8-cons
F_8516d4_S3-cons
F_8516d5_S4-cons
F_8516d6_S5-cons
F_8516d7_S6-cons
F_8530d4_S7-cons
F_8530d5_S8-cons
F_8531BALd5_S11-cons
F_8537d4_S9-cons
F_8537d5_S10-cons
8 9 10 11 12 13 14 4275 4276 4277 4278 4279 4280 4281 5239 5240 5241 5242 5243 5244 5245 6050 6051 6052 6053 6054 6055 6056 7071 7072 7073 7074 7075 7076 7077 8747 8748 8749 8750 8751 8752 8753 11509 11510 11511 11512 11513 11514 11515

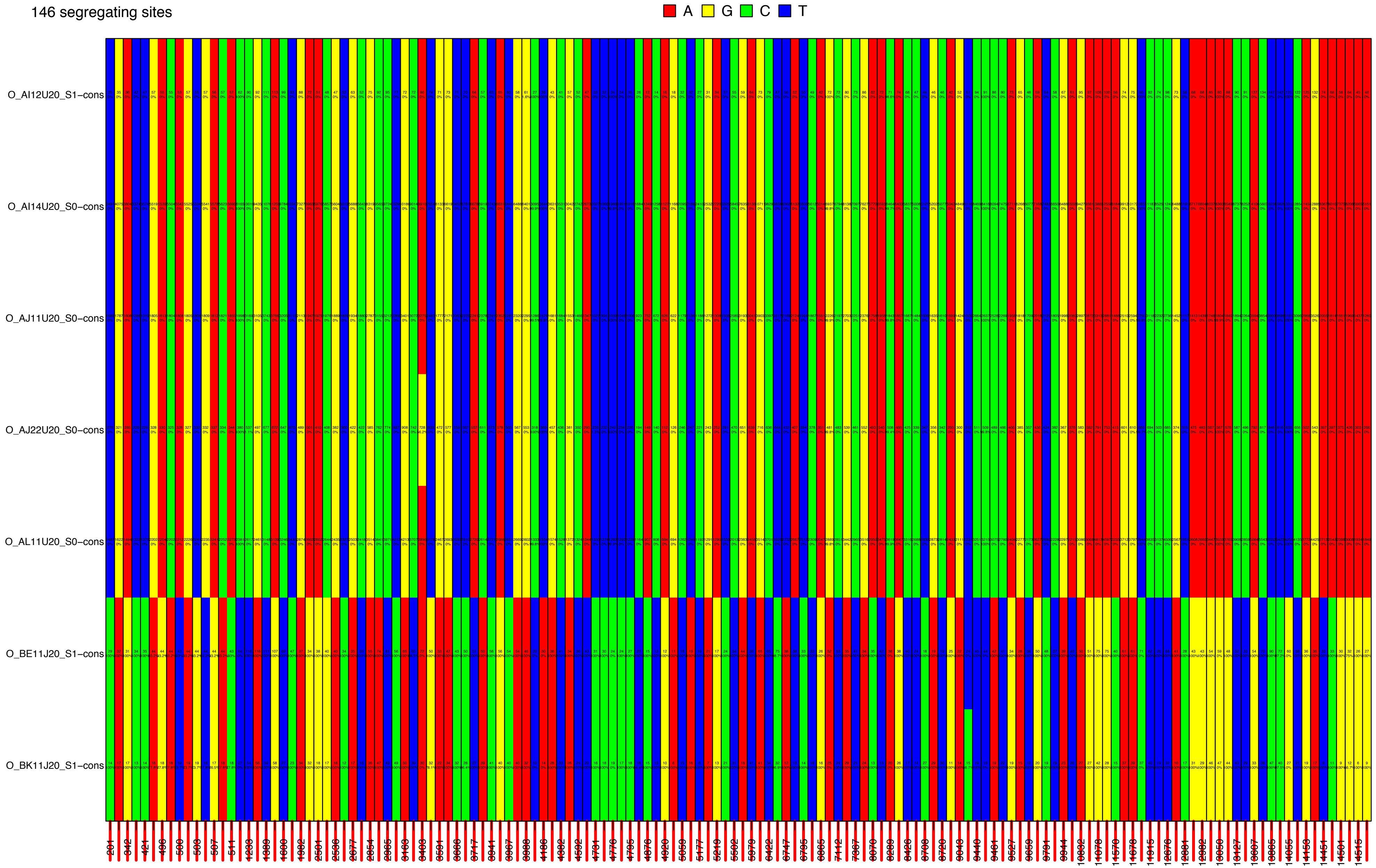
146 segregating sites
A
G
C
T
O_AI12U20_S1-cons
O_AI14U20_S0-cons
O_AJ11U20_S0-cons
O_AJ22U20_S0-cons
O_AL11U20_S0-cons
O_BE11J20_S1-cons
O_BK11J20_S1-cons
201
342
421
496
500
503
507
511
1233
1389
1600
1982
2501
2536
2677
2854
2965
3163
3403
3591
3666
3717
3941
3967
3988
4186
4382
4592
4731
4776
4795
4876
4920
5059
5177
5219
5502
5979
6422
6747
6795
6865
7112
7367
8070
8289
8426
8708
8720
9043
9440
9461
9527
9659
9791
9944
10832
11078
11570
11678
11915
12676
12881
12982
13050
13127
13607
13865
14055
14153
14451
14501
14515
